# RhoA determines lineage fate of mesenchymal stem cells by modulating CTGF–VEGF complex in extracellular matrix

**DOI:** 10.1038/ncomms11455

**Published:** 2016-04-29

**Authors:** Changjun Li, Gehua Zhen, Yu Chai, Liang Xie, Janet L. Crane, Emily Farber, Charles R. Farber, Xianghang Luo, Peisong Gao, Xu Cao, Mei Wan

**Affiliations:** 1 Department of Orthopaedic Surgery, Johns Hopkins University School of Medicine, Baltimore, Maryland 21205, USA; 2 Department of Public Health Sciences, Center for Public Health Genomics, University of Virginia, Charlottesville, Virginia 22908, USA; 3 Department of Endocrinology, Institute of Endocrinology and Metabolism, Second Xiangya Hospital of Central South University, Changsha 410011, China; 4 Department of Medicine, Johns Hopkins Asthma and Allergy Center, Johns Hopkins University School of Medicine, Baltimore, Maryland 21224, USA

## Abstract

Mesenchymal stem cells (MSCs) participate in the repair/remodelling of many tissues, where MSCs commit to different lineages dependent on the cues in the local microenvironment. Here we show that TGFβ-activated RhoA/ROCK signalling functions as a molecular switch regarding the fate of MSCs in arterial repair/remodelling after injury. MSCs differentiate into myofibroblasts when RhoA/ROCK is turned on, endothelial cells when turned off. The former is pathophysiologic resulting in intimal hyperplasia, whereas the latter is physiological leading to endothelial repair. Further analysis revealed that MSC RhoA activation promotes formation of an extracellular matrix (ECM) complex consisting of connective tissue growth factor (CTGF) and vascular endothelial growth factor (VEGF). Inactivation of RhoA/ROCK in MSCs induces matrix metalloproteinase-3-mediated CTGF cleavage, resulting in VEGF release and MSC endothelial differentiation. Our findings uncover a novel mechanism by which cell–ECM interactions determine stem cell lineage specificity and offer additional molecular targets to manipulate MSC-involved tissue repair/regeneration.

## Abstract (web summary)

It is unclear what regulates the fate of mesenchymal stem cells (MSCs) in arterial repair following injury. Here, the authors show that MSC differentiation following injury is triggered by RhoA which in turn stimulates the release of connective tissue growth factor and vascular endothelial growth factor.

## Graphical abstract

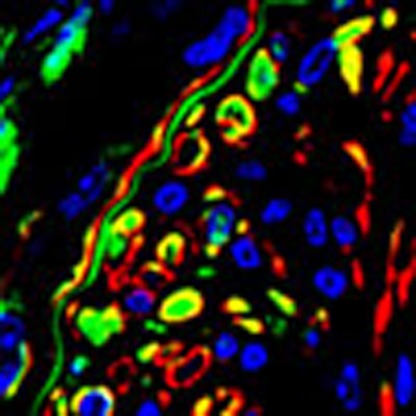

The ability of stem cells to differentiate to specific cell-matured phenotypes under defined conditions is termed ‘plasticity'^1^. Classically, the control of stem cell fate, has been primarily attributed to genetic and molecular mediators (for example, growth factors, transcription factors). Increasing evidence in the past two decades has revealed that the microenvironment is also a critical determinant for the lineage decision of stem cells. In particular, the ‘solid-state' environment, that is, the extracellular matrix (ECM), an essential component of stem cell microenvironment, constantly interacts with stem cells and regulates cell fate^2^,^3^,^4^. Stem cells produce and modify the ECM composition and topography. Conversely, dynamic changes in ECM regulate stem cell commitment/differentiation^3^,^5^,^6^. Mesenchymal stem cells (MSCs) are present in many types of tissues/organs and play a role in tissue repair/regeneration and pathological remodelling. Although *in vitro* evidence suggests that MSC–ECM interaction has a significant influence on the overall behaviour of the population, little is known on the molecular basis of specific MSC–ECM interactions during tissue repair/remodelling as well as the impact on MSC lineage specificity in a physiologic context.

Neointimal hyperplasia is classically believed to be the consequence of accumulated α-smooth muscle action (αSMA)-positive smooth muscle cells (SMCs) or myofibroblastic cells and the synthesis of ECM^7^,^8^. Neointimal hyperplasia plays a role in atherosclerosis, restenosis after angioplasty or bypass, diabetic vascular complications and transplantation arteriopathy. Specifically, in atherosclerotic vascular disease, neointima formation in the weeks and months after balloon angioplasty or stenting results in arterial restenosis with resultant morbidity and mortality^9^,^10^. Recent studies by our group and others suggest that a subpopulation of MSCs, specifically cells expressing nestin^11^, mobilize from their original niches to the vascular remodelling sites after arterial injury in mice^12^,^13^,^14^. Majority of the nestin^+^ cells recruited to the injured arteries gave rise to neointimal αSMA^+^ SMC/myofibroblastic cells^13^. Only a small portion of cells differentiated to the endothelial lineage for reendothelialization, which was shown to both promote physiologic endothelium repair and limit the neointima enlargement^15^,^16^,^17^.

Transforming growth factor β (TGFβ) has important roles in the development of the neointima and constrictive remodelling associated with angioplasty^18^,^19^. TGFβ is a multifunctional growth factor with effects on cell growth, differentiation, fibroblast activation and myofibroblast formation^20^,^21^, and ECM accumulation determined by downstream signalling events, such as the canonical Smad signalling pathways or noncanonical/alternative pathways (ERK, JNK, p38 MAPK, PI3K and RhoA/ROCK)^22^,^23^,^24^. For instance, we previously found that TGFβ signalling mediated via Smad signalling mobilizes nestin^+^ MSCs through peripheral blood to the injured artery^13^. Several recent studies demonstrated that TGFβ can also induce the differentiation of stem cells or progenitor cells towards SMC or myofibroblast lineage^25^,^26^.

In the present study, we delineated a molecular mechanism by which the lineage commitment/differentiation of nestin^+^ MSCs is controlled during vascular repair. Using a genetic nestin^+^ cell lineage mapping mouse model, we found that nestin^+^ cells recruited to the injured arteries is a contributor to neointimal formation. Nestin^+^ cells recruited to the remodelling sites represent a mixed population, with MSCs as a predominant component. These cells primarily differentiate into neointima SMCs/myofibroblastic cells through TGFβ-activated RhoA signalling. Inactivation of RhoA diverted the differentiation of nestin^+^ cells away from SMCs/myofibroblasts to endothelial cells for endothelium repair. Analysis the mechanisms underlying the MSC lineage shift revealed that MSCs with RhoA inactivation/inhibition secreted matrix metalloproteinase-3 (MMP3). MMP3 degraded the connective tissue growth factor (CTGF)–vascular endothelial growth factor (VEGF) ECM complex, releasing VEGF to promote endothelial differentiation. These findings provide a new understanding of the molecular basis by which the specification of MSC differentiation is regulated by local cues in the microenvironment to participate in tissue remodelling.

## Results

### Nestin^+^ cells at the injured arteries are predominantly MSCs

**Figure 1:**
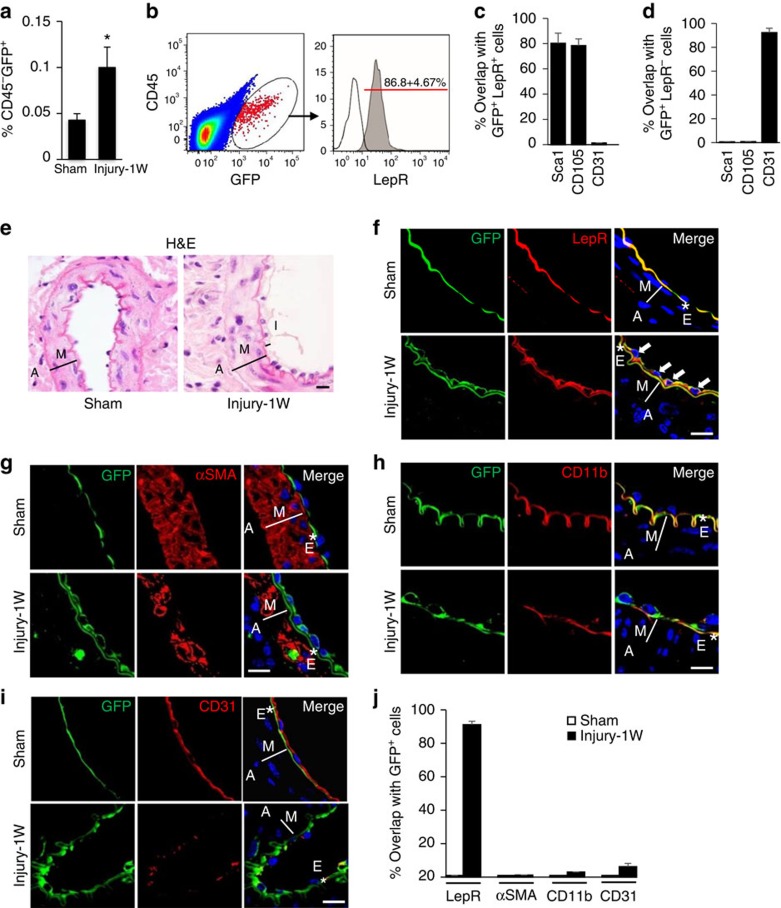
Nestin-GFP^+^ cell population at the arterial remodelling sites represents a subset of MSCs. *Nestin-GFP* mice were subjected to either sham surgery (Sham) or wire insertion-induced injury in femoral arteries (Injury). Blood samples were collected 1 week(1W) later. Percentages of blood CD45^−^ GFP^+^ cells out of total mononucleate cells from the mice were analysed by flow cytometry (**a**). *n*=5 mice per group. Data are represented as mean±s.e.m. *P<0.001 as determined by Student's *t*-tests. CD45^−^GFP^+^ cells were FACS sorted from circulating blood of the arterial injured mice (**b**, left panel). Representative image showing the percentage of the CD45^−^GFP^+^ cells that express LepR (**b**, right panel). The percentages of the GFP^+^LepR^+^ cells (**c**) or GFP^+^LepR^−^ cells (**d**) that express Sca1, CD105 or CD31, respectively, were analysed by flow cytometry. The data represent mean±s.e.m. from three to five mice from at least three independent experiments. *Nestin-GFP* mice were subjected to either sham surgery (Sham) or wire insertion-induced injury in femoral arteries (Injury). At 1W after the surgery, femoral arteries were harvested. Haematoxylin and eosin (H&E) staining of mouse femoral artery sections from the mice (**e**). A, adventitia layer; I, intima layer; M, media smooth muscle layer. Double-immunofluorescence images of femoral artery tissue sections from the mice using antibodies against GFP (green) and either LepR (**f**), αSMA (**g**), CD11b (**h**) or CD31 (**i**), respectively. *E, elastic fibre. White arrows represent double positive staining cells. 4,6-Diamidino-2-phenylindole stains nuclei blue. Scale bars, 50 μm. Quantification of the percentage of GFP^+^ cells that express LepR, αSMA, CD11b or CD31 at the injured sites (**j**). *n*=5. Data are represented as mean±s.e.m.

We previously demonstrated that nestin^+^ cells were mobilized to peripheral blood and recruited to the remodelling arteries as early as 1 week after arterial injury to participate in neointima formation^13^. Using a transgenic *Nestin-GFP* mouse model^27^, here we similarly found that non-haematopoietic CD45^−^GFP^+^ cells in peripheral blood of the mice increased more than twofold at 1 week after the mice were subjected to wire-induced injury of femoral artery (Fig. 1a). Our previous study revealed that the majority of mobilized nestin^+^ cells were Sca1^+^CD29^+^CD45^−^CD11b^−^ MSCs^13^,^14^. Here we further characterized the mobilized Nestin-GFP^+^ cells. Flow cytometry analysis revealed that after arterial injury, ∼87% of the circulating nestin-GFP^+^ cells expressed the leptin receptor (LepR; Fig. 1b), a recognized marker for adult bone marrow MSCs^28^,^29^. There are >80% GFP^+^LepR^+^ cells also expressed Sca1 or CD105, known cell surface markers expressed in stem/progenitor cells including MSCs^13^,^30^, whereas almost none (∼1.2%) of the GFP^+^LepR^+^ cells expressed CD31 (Fig. 1c). GFP^+^LepR^+^ cells were capable of osteogenesis (Supplementary Fig. 1a), adipogenesis (Supplementary Fig. 1b) and chondrogenesis (Supplementary Fig. 1c). Furthermore, these cells have abilities to differentiate into CD31^+^ endothelial-like cells (Supplementary Fig. 1d) and αSMA^+^ smooth muscle like cells/myofibroblasts (Supplementary Fig. 1e). On the contrary, the majority of the GFP^+^LepR^−^ cell population were CD31^+^ (∼92%), Sca1^−^ and CD105^−^ (Fig. 1d) and did not possess multi-differentiation potential (data not shown), suggesting that GFP^+^LepR^−^ cells are endothelial lineage cells. Thus, increased circulating nestin^+^ cells in response to arterial injury represent a mix population of cells with MSCs as a predominant component.

Nestin-GFP^+^ cells were further characterized by *in situ* immunofluorescence staining after vessel injury. Haematoxylin and eosin and immunofluorescence staining demonstrated that at 1 week after arterial injury, Nestin-GFP^+^ cells formed a single intraluminal layer (Fig. 1e–i) and the majority of the GFP^+^ cells (>90%) recruited to the injured arterial sites expressed LepR (Fig. 1f,j), but rarely expressed αSMA (∼0.6%), CD11b (∼3.2%) or CD31 (∼6.4%; Fig. 1g–j). The results suggest that Nestin-GFP^+^ cells recruited to the injured arterial sites are predominantly MSCs.

To obtain direct evidence for the involvement of circulating nestin^+^ cells in neointima formation, we generated heterochronic parabiosis mouse pairs. In the mouse pairs, a wild-type (WT) mouse was surgically conjoined to a *Nestin-cre; ROSA26-EYFP* mouse (Supplementary Fig. 2a). In the *Nestin-cre; ROSA26-EYFP* mice both nestin^+^ cells and their descendants can be detected of EYFP fluorescence. At 2 weeks post surgery, partner-derived EYFP^+^ cells were detected in the blood from the WT parabiont. Blood EYFP^+^ cells in WT parabiont represented ∼49% of the total EYFP^+^ cells in the blood from the Nes-R26-EYFP isochronic parabiotic pair (Supplementary Fig. 2b, compare the 4th Bar with the 2nd Bar), indicating a cross circulation was established and blood chimerism was formed. Although EYFP^+^ cells were not detected in the uninjured arteries in the WT parabiont (Supplementary Fig. 2c, upper panel, and Supplementary Fig. 2d), many partner-derived EYFP^+^ cells (∼22.5% of total neointimal cells) were detected in the injured arteries of the WT parabiont (Supplementary Fig. 2c, bottom panel, and Supplementary Fig. 2d). Thus, blood circulation-derived nestin^+^ cells contribute to the both neointimal hyperplasia and vascular healing after arterial injury.

### Nestin^+^ cells differentiate into neointimal myofibroblasts

**Figure 2:**
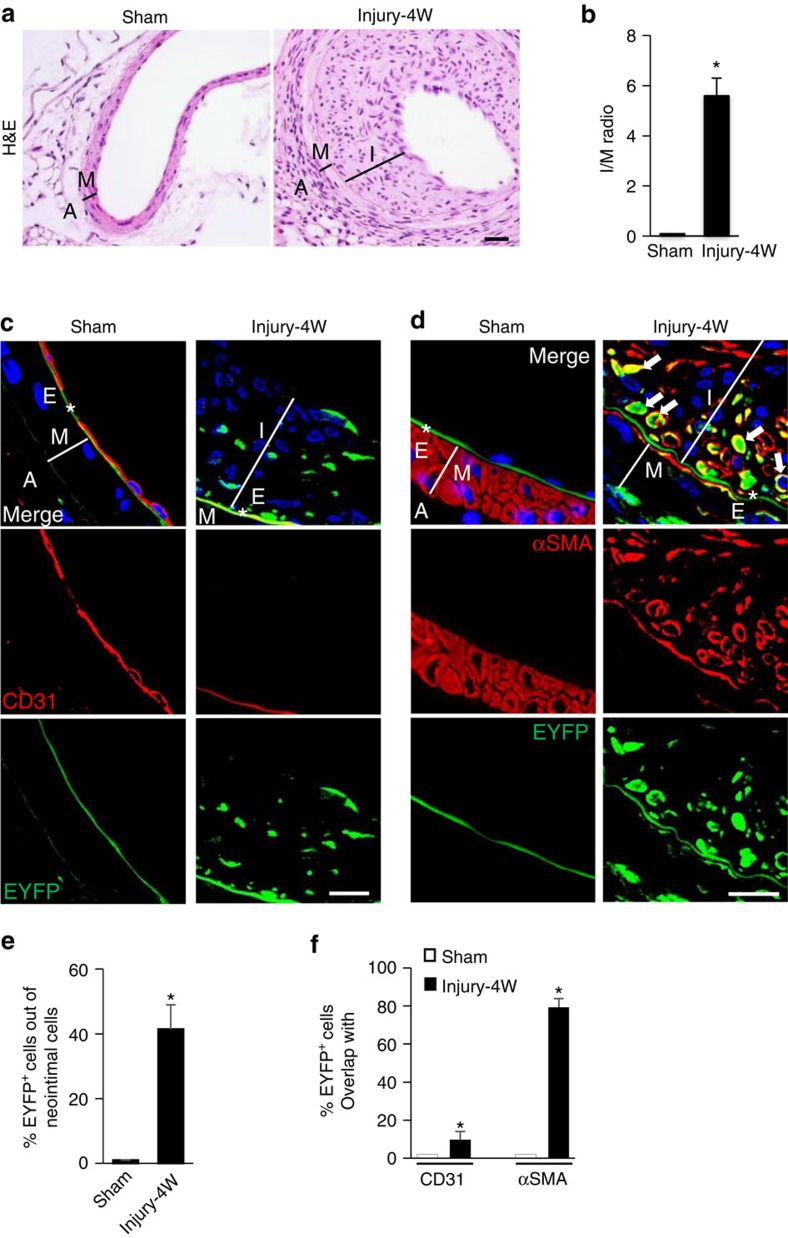
Nestin^+^ cells predominantly give rise to neointimal SMCs/myofibroblasts at the remodelling sites of injured arteries. *Nestin-Cre::ROSA26-EYFP* mice were subjected to either sham surgery (Sham) or wire insertion-induced injury in femoral arteries (Injury). At 4 weeks (4W) after the surgery, femoral arteries were harvested. (**a**) Haematoxylin and eosin (H&E) staining of mouse femoral artery sections from the mice. A, adventitia layer; I, intima layer; M, media smooth muscle layer. (**b**) Ratio of intima/media areas (*I*/*M* ratio) of the mouse femoral arteries. *n*=5 mice per group. Double-immunofluorescence images of tissue sections of mouse femoral artery using antibodies against EYFP and CD31 (**c**) or αSMA (**d**), respectively. *E, elastic fibre. White arrows represent double-positive staining cells. 4,6-Diamidino-2-phenylindole stains nuclei blue. Scale bars, 50 μm. (**e**) Percentage of EYFP^+^ cells out of total neointimal cells from Sham or Injury group. *n*=5. (**f**) Percentage of EYFP^+^ cells from Sham or Injury group that express CD31 or αSMA. *n*=5. Data are represented as mean±s.e.m. *P<0.001 versus Sham as determined by Student's *t*-tests.

We then tracked the lineage fate of the newly recruited nestin^+^ cells at the injured sites of arteries using *Nestin-cre; ROSA26-EYFP* mice. Examination of the fate of Nestin-EYFP^+^ cells 4 weeks after arterial injury, when neointima tissue was very thick (Fig. 2a,b), revealed that ∼41% of the neointimal cells were EYFP^+^ (Fig. 2c–e). Notably, the majority of EYFP-labelled cells were αSMA positive (Fig. 2d,f) and CD31 negative (Fig. 2c,f) in the neointima tissue in the injured arteries. However, in the uninjured sham-operated arteries, CD31^+^ endothelial cells formed an intact intraluminal layer (Fig. 2c). Thus, nestin^+^ cells involved in arterial remodelling after endoluminal injury mainly gave rise to neointimal SMCs/myofibroblasts.

### RhoA activation induces MSC differentiation to myofibroblasts

**Figure 3:**
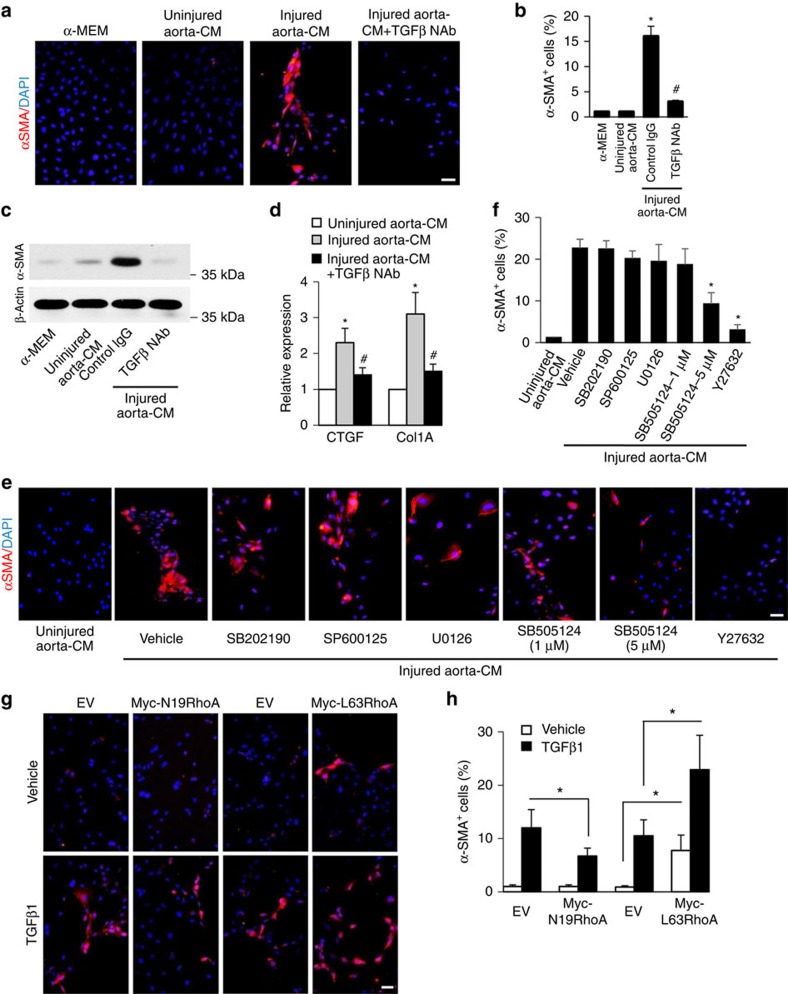
RhoA/ROCK is a key signalling pathway for the differentiation of MSCs to SMCs/myofibroblasts during arterial remodelling. Mouse MSCs were incubated in αMEM (control medium), uninjured aorta-CM, injured aorta-CM with or without TGFβ-neutralizing antibody (TGFβ NAb) for 7 days. Immunofluorescence staining of MSCs using antibody against αSMA (**a**, shown in red). 4,6-Diamidino-2-phenylindole (DAPI) stains nuclei blue. Percentage of total cells expressing αSMA per × 20 magnification field (**b**). *n*=4, *P<0.001 versus uninjured aorta-CM, ^#^*P*<0.01 versus injured aorta-CM+control IgG. Western blot analysis of αSMA or β-actin expression in MSCs with different treatment (**c**). Quantitative real-time PCR analysis of connective tissue growth factor (CTGF) and collagen type 1 (Col1A) in MSCs with different treatment (**d**). *n*=5; *P<0.01 versus uninjured aorta-CM group; ^#^*P*<0.01 versus injured aorta-CM+control IgG as determined by ANOVA. Mouse MSCs were incubated with uninjured aorta-CM or injured aorta-CM with addition of either vehicle or specific inhibitors as indicated for 7 days. Immunofluorescence staining of MSCs using antibody against αSMA (**e**). Percentage of total cells expressing α-SMA^+^ per × 20 magnification field (**f**). *n*=4, *P<0.01 versus injured aorta-CM+vehicle determined by ANOVA. Myc-N19RhoA, Myc-L63RhoA or empty vector (EV) was transfected into mouse MSCs and treated with either vehicle or 5 ng ml^−1^ TGFβ1 for 7 days. Immunofluorescence staining of the cells with antibody against αSMA (**g**). Percentage of total cells expressing α-SMA per × 20 magnification field (**h**). Scale bars, 100 μm. *n*=5. Data are represented as mean±s.e.m. *P<0.001 as determined by ANOVA.

To identify the molecular signalling pathway involved in MSC differentiation after arterial injury, we developed an injured aorta-conditioned medium (CM)-based differentiation assay, in which mouse MSCs were incubated in CM prepared from *ex vivo* injured aorta for 7 days^13^. Nearly 100% of these MSCs express nestin (Supplementary Fig. 3). CM prepared from *ex vivo* injured aorta stimulated the differentiation of Nestin^+^ MSCs into SMCs/myofibroblasts detected by both immunofluorescence staining (Fig. 3a,b) and western blot analysis (Fig. 3c) using specific antibody against αSMA, a marker of SMCs/myofibroblasts. CM prepared from uninjured aorta did not have such effect. TGFβ has shown to induce the differentiation of stem cells towards SMC or myofibroblast lineage^25^,^26^. As expected, when neutralizing antibody against TGFβ1 (TGFβ1 NAb) was added to the injured aorta-CM, the differentiation of MSCs was abolished (Fig. 3a–c). CTGF, an enhancer of ECM deposition and collagen type 1A (Col1A), a component of ECM, are made by fibroblasts/myofibroblasts. Consistently, the mRNA levels of both CTGF and Col1A, were elevated in cells incubated with injured aorta-CM relative to cells incubated with uninjured aorta-CM, whereas addition of TGFβ1 NAb blocked the elevation of these two genes (Fig. 3d). Moreover, ECM formation was largely promoted by MSCs incubated with injured aorta-CM as indicated by increased fibronectin and collagen I expression (Supplementary Fig. 4). These results confirm that active TGFβ1 released from injured arteries is a key factor for the differentiation of nestin^+^ MSCs towards SMCs/myofibroblasts.

The role of canonical and noncanonical TGFβ signalling pathways in mediating MSC differentiation stimulated by injured aorta-CM were detected by adding individual inhibitors of various signalling pathways known to be activated by TGFβ. Addition of Y27632 (RhoA/ROCK inhibitor) in the injured aorta-CM blocked the differentiation of MSCs into αSMA^+^ SMCs/myofibroblasts, whereas SMC differentiation was not affected by adding SB202190 (p38 inhibitor), SP600125 (JNK inhibitor) or U0126 (ERK inhibitor) in the injured aorta-CM (Fig. 3e,f). Interestingly, a higher dose (5 μM) of SB505124 (TβRI/Smad inhibitor), which showed potent inhibitory effects on both Smad phosphorylation and RhoA activation (Supplementary Fig. 5), inhibited MSC differentiation, whereas a lower dose (1 μM) of SB505124, which only inhibited Smad2 phosphorylation (Supplementary Fig. 5), failed to inhibit MSC differentiation (Fig. 3e,f). Therefore, TGFβ appeared to stimulate MSC differentiation primarily through a RhoA-dependent pathway. Consistently, double-immunofluorescence analysis of the arterial sections from *Nestin-GFP* mice revealed at 1 weeks after the injury of femoral arteries, the majority of nestin-GFP^+^ cells in neointimal tissue expressed active RhoA-GTP (Supplementary Fig. 6).

We further tested if manipulating the activity of RhoA in nestin^+^ MSCs would change the differentiation of the cells by individually expressing a constitutively active RhoA (L63RhoA) or a dominant negative RhoA (N19RhoA) in mouse MSCs (Supplementary Fig. 7). Cells were then treated with TGFβ1 for 7 days to activate endogenous RhoA. TGFβ1 stimulated the differentiation of empty vector (EV)-transfected MSCs towards SMC/myofibroblastic cells as indicated by increased αSMA^+^ cells (Fig. 3g,h). Knockdown of RhoA resulted in decreased SMC differentiation, whereas overexpression of RhoA resulted in a significant increase in the number of MSCs that differentiated into αSMA^+^ cells relative to EV-transfected control cells, regardless of the presence or absence of TGFβ1 (Fig. 3g,h; analysis of variance (ANOVA)), indicating that RhoA activation itself can stimulate MSC differentiation. Taken together, the results suggest that RhoA/ROCK signalling contributes to pathological intimal hyperplasia in the injured arteries by promoting SMCs/myofibroblasts differentiation of MSCs.

### Inactivation of RhoA changes the lineage fate of MSCs

**Figure 4:**
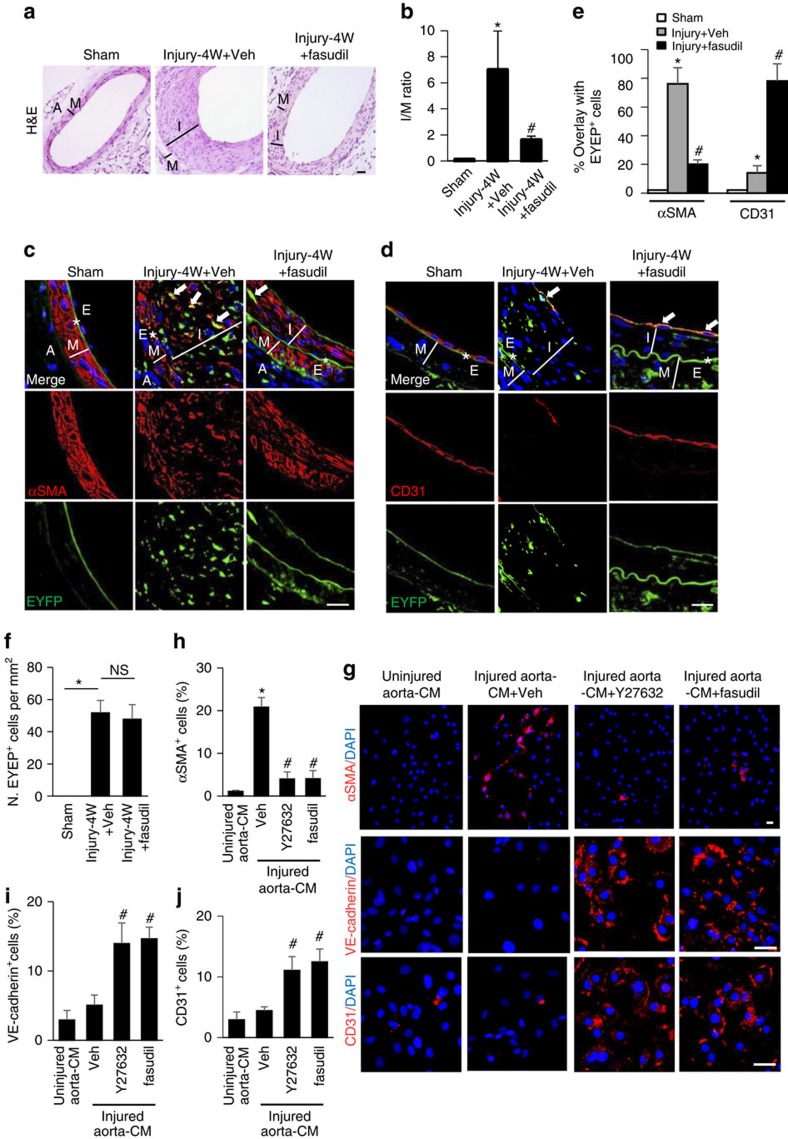
RhoA/ROCK inhibitor promotes endothelium repair and inhibits neointima formation in mice with arterial injury. *Nestin-Cre::ROSA26-EYFP* mice were subjected to either sham surgery or wire insertion-induced injury in femoral arteries 4 weeks (4W) after procedure. Fasudil at a dose of 30 mg kg^−1^ dissolved in water was administered by an oral gavage twice daily to mice, starting at 3 days before the surgery until 4W after. (**a**) Haematoxylin and eosin (H&E) staining of the artery sections from the mice. A, adventitia layer; I, intima layer; M, media smooth muscle layer. (**b**) Ratio of intima/media areas (*I*/*M* ratio) of mouse femoral arteries. *n*=5, *P<0.001 versus Sham. ^#^*P*<0.001 versus Injury+Veh (vehicle) group determined by ANOVA. Double-immunofluorescence images of EYFP (green) and αSMA (red; **c**) or CD31 (red; **d**) of the artery sections. *E, elastic fibre. White arrows represent double-positive staining cells. 4,6-Diamidino-2-phenylindole (DAPI) stains nuclei blue. Scale bars, 50 μm. (**e**) Percentage of EYFP^+^ cells expressing either αSMA or CD31 in arteries from different treatment group. *n*=5, *P<0.001 versus Sham. ^#^*P*<0.001 versus Injury+Veh group determined by ANOVA. (**f**) Quantification of the number of EYFP^+^ cells per tissue area (N. EYFP^+^cell per mm^2^). *n*=5, *P<0.01; NS, not significant, as determined by ANOVA. Mouse MSCs treated with uninjured aorta-CM, injured aorta-CM, injured aorta-CM+Y27632 or injured aorta-CM+fasudil for 7 days. Immunofluorescence staining of the cells using individual antibodies against αSMA, VE-cadherin, or CD31 (**g**). Scale bars, 50 μm. Quantification of the percentages of cells positive for αSMA (**h**), VE-cadherin (**i**), and CD31 (**j**) out of total cells. n=5. Data are represented as mean±s.e.m. *P<0.001 versus Uninjured Aorta-CM. ^#^*P*<0.001 versus injured Aorta-CM+Veh as determined by ANOVA.

To validate the role of RhoA/ROCK in the lineage differentiation of nestin^+^ MSCs and neointima formation *in vivo*, we examined if the ROCK inhibitor fasudil could inhibit the differentiation of MSCs towards SMCs/myofibroblasts and reduce neointima formation. Treating the *Nestin-Cre::ROSA26-EYFP* mice with fasudil reduced the formation of neointima (Fig. 4a,b) and EYFP-labelled cells in the deeper layers of neointimal tissue (Fig. 4c,d). Further, fasudil treatment dramatically decreased EYFP-labelled αSMA^+^ SMCs/myofibroblasts in neointimal tissue of the arterial injured mice relative to vehicle-treated mice (Fig. 4c–e). Surprisingly, a full layer of CD31^+^ endothelial cells were seen at the intra-luminal side of neointima tissue in mice with fasudil treatment (Fig. 4d). Moreover, ∼78% of the CD31^+^ cells of the repaired endothelium were co-labelled with EYFP (Fig. 4d,e), whereas only ∼14% of the EYFP-labelled cells in neointima tissue of the vehicle-treated mice expressed CD31 (Fig. 4d,e). The number of the total EYFP^+^ cells (including αSMA^+^ SMCs/myofibroblasts and CD31^+^ cells) within the neointimal tissue in mice with fasudil treatment was not different compared with vehicle-treated group, indicating that the recruitment of the EYFP^+^ cells were not affected by fasudil (Fig. 4f). Thus, inactivation of RhoA/ROCK not only reduced the differentiation of nestin^+^ MSCs to SMCs/myofibroblastic lineage but also instead promoted their differentiation towards endothelial cell lineage during arterial remodelling.

To further test the hypothesis that inactivation of RhoA/ROCK signalling during arterial remodelling may change the lineage fate of the recruited MSCs, we evaluated the effect of RhoA inhibition in our injured aorta-CM-based cell differentiation assay. About 20.4% of MSCs differentiated into αSMA^+^ SMCs/myofibroblasts after exposure to injured aorta-CM. Addition of the ROCK inhibitor Y27632 or fasudil reduced the MSC differentiation into SMCs/myofibroblast (Fig. 4g, upper panels, and 4 h) and instead resulted in MSC differentiation into VE-cadherin^+^ (13.9 and 14.6%) and CD31^+^ (11.2 and 12.5%) endothelial cells (Fig. 4g, middle and bottom panels, and 4i and 4j). Collectively, the *in vivo* and *in vitro* results suggest that inactivation of RhoA/ROCK not only inhibits MSC myofibroblast differentiation and neointimal hyperplasia but also triggers the switch of MSCs to participate in endothelium repair after arterial injury (Supplementary Fig. 8).

### RhoA regulates VEGF release from ECM protein complex

**Figure 5:**
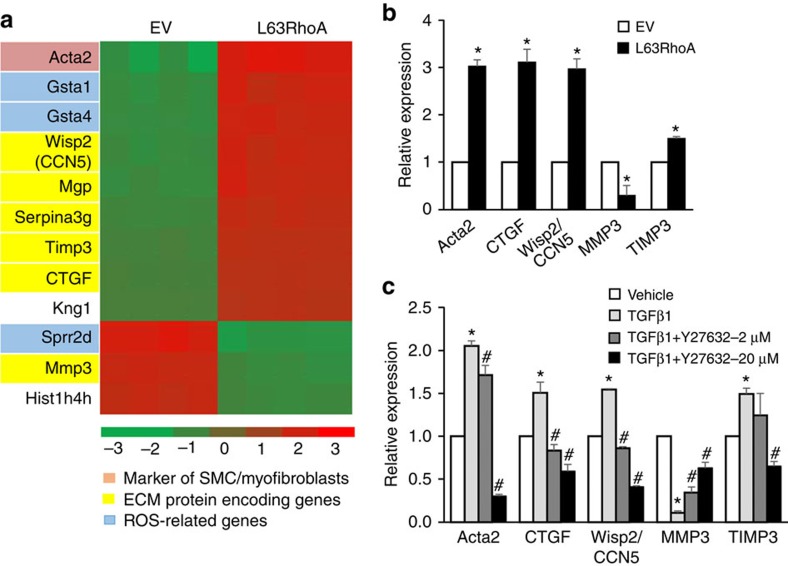
Extracellular matrix (ECM) genes are differentially expressed in MSCs expressing active RhoA. Mouse MSCs were transfected with either empty vector (EV) or L63RhoA. Heat map of the 12 genes identified as differentially expressed by microarray analysis (**a**, fold change >2, false-discovery rate<0.20). Validation of the mRNA changes of the indicated genes by qRT–PCR analysis (**b**). *n*=5, *P<0.01 versus EV group as determined by Student's *t*-tests. (**c**) Mouse MSCs were treated with 5 ng ml^−1^ TGFβ1 alone or in combination with increasing doses of Y27632 for 7 days. qRT–PCR analysis of the indicated genes in MSCs. *n*=5. Data are represented as mean±s.e.m. *P<0.01 versus vehicle group. ^#^*P*<0.01 versus TGFβ1+Veh group as determined by ANOVA.

To identify the downstream molecules that mediate RhoA-induced MSC differentiation, we examined cDNA isolated from mouse MSCs with L63RhoA expression using a whole-genome expression microarray. A total of 12 differentially expressed genes were identified in MSCs transfected with L63RhoA relative to control cells, with 9 genes upregulated and 3 downregulated (Fig. 5a). Among these genes, two specific marker genes of functional myofibroblasts Acta2 (αSMA encoding gene; Fig. 5a, in pink) and CTGF were identified. In addition to CTGF, an array of ECM proteins encoding genes including CCN5/Wisp2, MMP3 and Timp3 were differentially expressed in cells transfected with L63RhoA relative to the control cells transfected with EV. Further quantitative reverse transcription–PCR (qRT–PCR) analysis confirmed the changes of these genes in L63RhoA-expressing cells compared with control cells (Fig. 5b). We then determined if the same genes were significantly changed in MSCs treated with TGFβ1 relative to cells treated with vehicle, the mRNA levels of Acta2, CTGF, CCN5/Wisp2 and Timp3 were upregulated, whereas MMP3 levels were dramatically downregulated (approximately ninefold decrease versus control group) in TGFβ1-treated cells. Moreover, RhoA/ROCK inhibitor Y27632 dose-dependently reversed the changes of these genes induced by TGFβ1 (Fig. 5c). The results suggest that the effect of RhoA on determining the lineage differentiation of MSCs may be through modulation of ECM remodelling.

**Figure 6:**
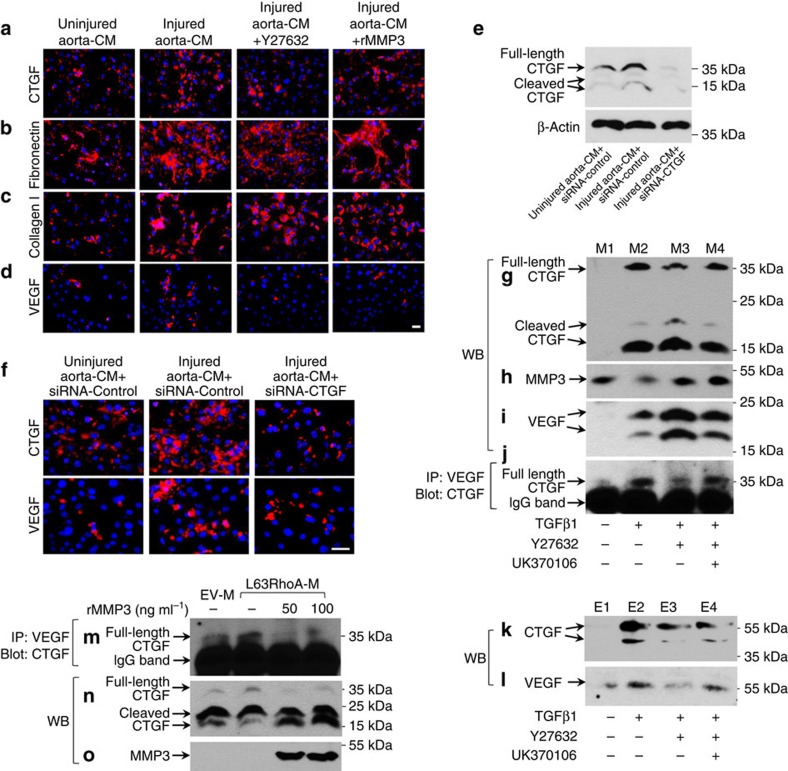
Activation of RhoA promotes CTGF–VEGF complex formation in ECM, whereas inactivation of RhoA induces MMP3-mediated CTGF cleavage and VEGF release. (**a**–**d**) Immunofluorescence analysis of the binding of VEGF to ECM in different MSC cultures. MSCs were incubated with the indicated treatments for 7 days. Immunofluorescence staining was performed on non-permeabilized cells using antibodies against CTGF (**a**), fibronectin (**b**), collagen I (**c**) or VEGF (**d**). (**e**,**f**) MSCs were transfected with siRNA-control or siRNA-CTGF, then cultured with the indicated medium for 6 days. Western blot (WB) analysis of the cell lysates was performed using antibodies against CTGF and β-actin (**e**). Immunofluorescence staining was performed on non-permeabilized cells using antibodies against CTGF or VEGF (**f**). (**g**–**j**) WB and co-immunoprecipitation (IP) analyses of VEGF and CTGF in the overlay media of MSCs with different treatment. MSCs were incubated with the indicated treatments for 7 days. Overlay media: M1, M2, M3 and M4 were collected, respectively. Western blotting analysis of the overlay media was performed using antibodies against CTGF (**g**), MMP3 (**h**) and VEGF (**i**). Overlay media were subjected to IP assays using antibody against VEGF, the VEGF-associated CTGF was detected by western blotting with antibody against CTGF (**j**). (**k**,**l**) WB analysis of VEGF and CTGF in the ECMs of the cultured MSCs with different treatment. MSCs were incubated with the indicated treatment for 7 days. ECMs: E1, E2, E3 and E4 were collected. Western blotting analysis of the ECMs was performed using antibodies against CTGF (**k**) and VEGF (**l**). (**m**–**o**) WB and co-IP analysis of VEGF and CTGF in the overlay media of MSCs transfected with empty vector (EV) or L63RhoA. In the aliquots of the overlay medium collected from the cells overexpressed L63RhoA, 50 or 100 ng ml^−1^ rMMP3 was added and the reactions were maintained at 37 °C for 2 h. Overlay media were subjected to immunoprecipitation assays using antibody against VEGF, the VEGF-associated CTGF was detected by western blotting with antibody against CTGF (**m**). Western blotting analysis of the overlay media was performed using antibodies against CTGF (**n**) and MMP3 (**o**). Scale bars, 50 μm. Data are representative of three independent experiments.

It has been reported that ECM-bound growth factors or cytokines can be released via MMP-mediated proteolytic mechanisms^31^. CTGF is a matricellular protein that binds many growth factors, including VEGF^32^,^33^,^34^. As we found a dramatic increase in MMP3 level in MSCs with RhoA/ROCK inhibition, we postulated that increased MMP3 may degrade CTGF and disrupt CTGF–VEGF binding in ECM, resulting in VEGF release for endothelial differentiation of MSCs. To test the hypothesis, we first assessed the binding of VEGF to ECM in the MSC culture. MSCs treated with injured-aorta CM promoted CTGF production (Fig. 6a) and ECM formation as indicated by increased expressions of fibronection (Fig. 6b) and collagen I (Fig. 6c). VEGF bound to the cell monolayer and ECM was also increased as detected by immunofluorescence analysis of VEGF level in the non-permeabilized cells that were incubated with injured-aorta CM relative to control CM-treated cells (Fig. 6d). Addition of either a RhoA/ROCK inhibitor Y27632 or recombinant MMP3 (rMMP3) reduced the ECM-bound VEGF elicited by injured-aorta CM without affecting the expression of fibronection and collagen I. Consistently, knockdown of CTGF by small interfering RNA (siRNA; Fig. 6e) also reduced the binding of VEGF to the cell monolayer and ECM (Fig. 6f), indicating the opposite roles of CTGF and MMP3 for VEGF sequestration and release.

To assess the direct binding of CTGF and VEGF in ECM, western blot analysis and co-immunoprecipitation assays were performed. The overlay media and ECM were collected separately from four different MSC cultures: α-minimum essential medium (αMEM) with 1% serum only (M1 and E1), adding TGFβ1 in the culture medium (M2 and E2), adding both TGFβ1 and RhoA/ROCK inhibitor Y27632 in the culture medium (M3 and E3), or adding TGFβ1, Y27632 and a specific MMP3 inhibitor UK370106 in the culture medium (M4 and E4). Expression of full-length CTGF (about 37 kDa) and smaller fragments (15–25 kDa) was markedly induced by TGFβ1 in the overlay medium of MSC culture as compared with the vehicle-treated cells (Fig. 6g, M2 versus M1). Similarly, strong expression of CTGF was also detected in the ECM prepared from TGFβ1-treated cells (Fig. 6k, E2 versus E1). Consistent with the gene array data in Fig. 5, TGFβ1 reduced MMP3 level in the overlay medium (Fig. 6h, M2 versus M1). Addition of Y27632 decreased the level of full-length CTGF but increased levels of smaller fragments of CTGF in the overlay medium (Fig. 6g, M3 versus M2), suggesting a cleavage of CTGF in response to RhoA inhibition of MSCs. Importantly, more VEGF protein was seen in the overlay medium (Fig. 6i, M3 versus M2), whereas less VEGF was detected in ECM (Fig. 6l, E3 versus E2) when the cells were exposed to TGFβ1+RhoA inhibitor relative to TGFβ1 alone, indicating VEGF release from ECM to the medium. Addition of a MMP3 inhibitor abrogated these effects (Fig. 6i, M4 versus M3, and Fig. 6l, E4 versus E3). Co-immunoprecipitation assays showed stronger binding of VEGF to CTGF in TGFβ1-treated overlay medium relative to control medium (Fig. 6j, M2 versus M1). Adding Y27632 reduced CTGF–VEGF binding (Fig. 6j, M3 versus M2). Further addition of a specific MMP3 inhibitor restored the binding (Fig. 6j, M4 versus M3), indicating the disruption of CTGF–VEGF complex by RhoA inhibition-induced MMP3 upregulation. In a similar manner, overexpressing constitutive active RhoA (L63RhoA) in MSCs induced the complex formation of CTGF and VEGF in the overlay medium (Fig. 6m, Lane 2), and adding rhMMP3 abolished CTGF–VEGF complex formation (Fig. 6m, 3rd and 4th lane). Moreover, rhMMP3 effectively cleaved CTGF into smaller fragments (Fig. 6n, 3rd and 4th lane). Together, the results suggest that TGFβ-induced secretion of VEGF from MSCs is inactivated by binding to CTGF in ECM, and MMP3 produced by MSCs with RhoA inactivation proteolytically degrades CTGF and releases VEGF to permit its angiogenic function.

### RhoA inhibition promotes endothelial differentiation of MSCs

**Figure 7:**
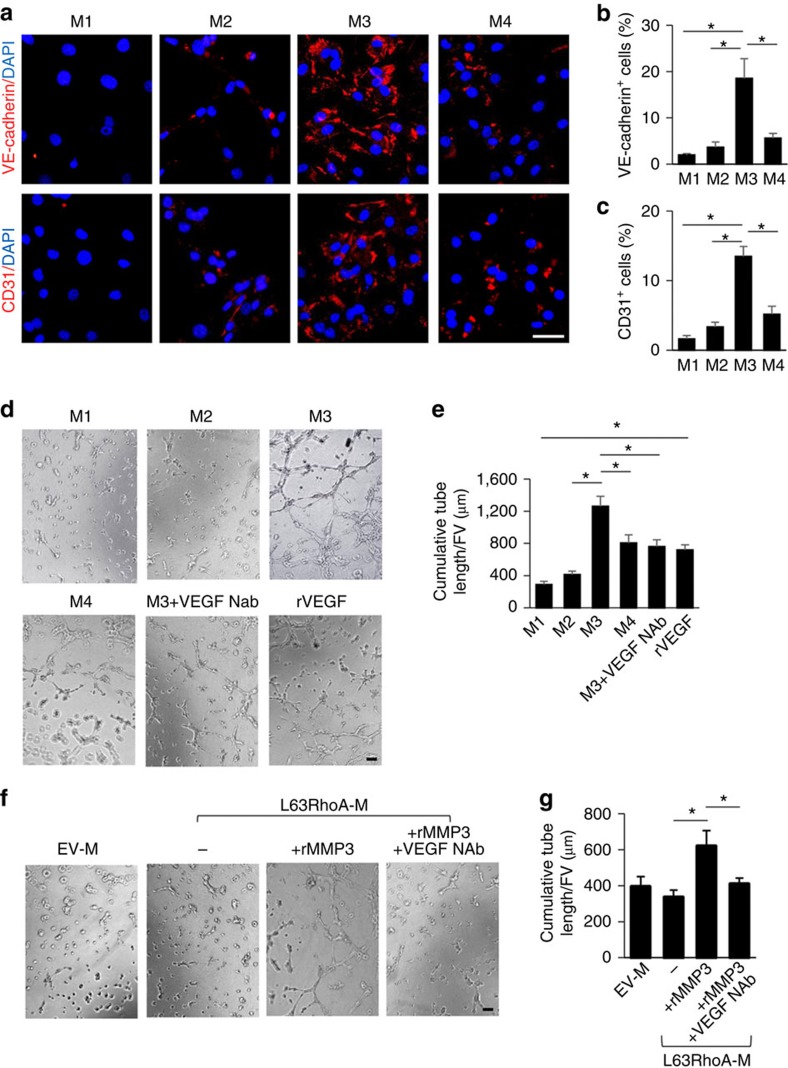
Inactivation of RhoA promotes endothelial differentiation of MSCs through MMP3. Mouse MSCs were incubated with different media: αMEM with 1% serum only (M1), adding TGFβ1 (M2), adding both TGFβ1 and RhoA/ROCK inhibitor Y27632 (M3) or adding TGFβ1, Y27632 and a specific MMP3 inhibitor UK370106 (M4) for 6 days. Immunofluorescence staining of the cells using individual antibodies against VE-cadherin and CD31 (**a**). Quantification of the percentages of VE-cadherin- (**b**) and CD31- (**c**) positive staining cells out of total cells. *n*=5. Scale bars, 50 μm. Representative images of tube formation (**d**) and quantitative data of cumulative tube length (**e**) of MSCs cultured with indicated overlay medium (see the description in **a**). rVEGF treatment serves as a positive control. *n*=5. Representative images of tube formation (**f**) and quantitative data of cumulative tube length (**g**) of MSCs cultured with indicated overlay medium. *n*=5. Scale bars, 200 μm. Data are represented as mean±s.e.m. *P<0.01 as determined by ANOVA (**b**,**e**,**g**). DAPI, 4,6-diamidino-2-phenylindole.

Although VEGF was moderately increased in the medium from TGFβ1-treated MSCs relative to control cells, the medium only modestly induced the differentiation of MSCs into CD31^+^,VE-caherin^+^ endothelial cells (Fig. 7a–c) and tube formation (Fig. 7d,e). On the other hand, medium collected from the MSC culture with concomitant treatment of TGFβ1 and RhoA/ROCK inhibitor had dramatic effects on both endothelial differentiation (Fig. 7a–c) and tube formation (Fig. 7d,e), which was abolished by adding either MMP3 inhibitor or anti-VEGF antibody. Medium collected from MSCs transfected with active RhoA and treated with rMMP3 also elicited angiogenic activity, which was abolished by adding anti-VEGF antibody (Fig. 7f,g). Thus, inactivation of RhoA induces CTGF degradation and VEGF activation, promoting endothelial differentiation of MSCs.

### RhoA inhibitor increases VEGF signalling in the injured arteries

**Figure 8:**
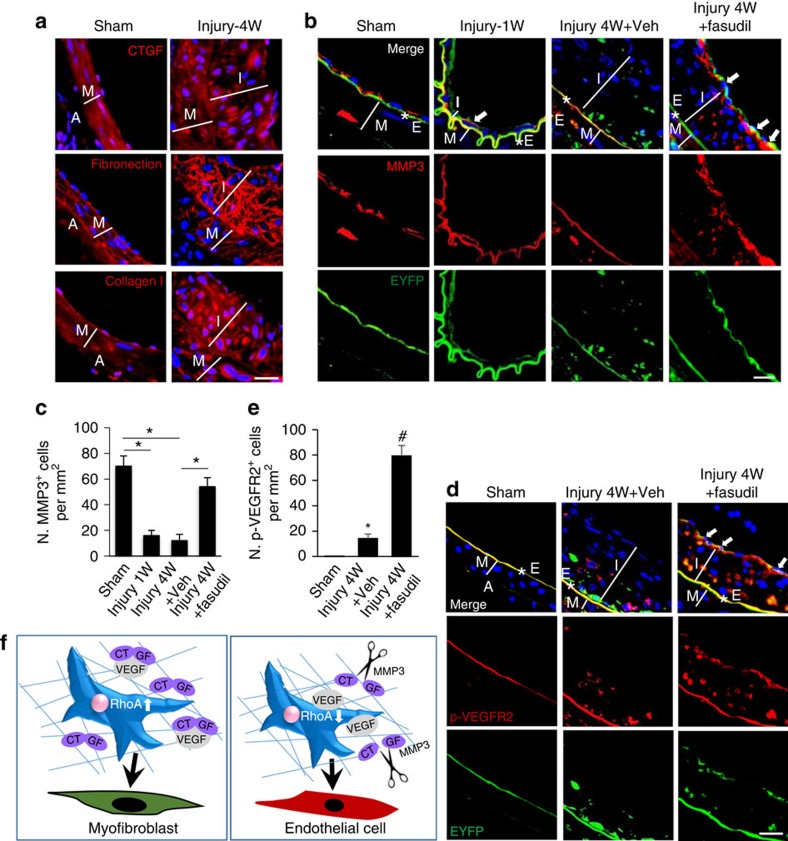
Increased MMP3 in ECM and VEGF signalling in EYFP^+^ cells were seen at the intima layer of the injured arteries in mice treated with ROCK inhibitor. *Nestin-Cre::ROSA26-EYFP* mice were subjected to either sham surgery or wire insertion-induced injury in femoral arteries 4 weeks (4W) after procedure. Fasudil at a dose of 30 mg kg^−1^ dissolved in water was administered by an oral gavage twice daily to mice, starting at 3 days before the surgery until 4 weeks after. Immunofluorescence staining of the artery sections using antibodies against CTGF, fibronectin and collagen I (**a**, in red). Double-immunofluorescence staining of the artery sections using antibodies against EYFP (green) and MMP3 (red) (**b**). *E, elastic fibre. White arrows represent double-positive staining. 4,6-Diamidino-2-phenylindole stains nuclei blue. Scale bars, 50 μm. Quantification of the number of MMP3^+^ cells per tissue area (N. MMP3^+^ cell per mm^2^; **c**). *n*=5. Data are represented as mean±s.e.m. *P<0.01 determined by ANOVA. Double-immunofluorescence staining of the artery sections using antibodies against EYFP (green) and p-VEGFR2 (red) (**d**). A, adventitia layer; I, intima layer; M, media smooth muscle layer; *E, elastic fibre. White arrows represent double-positive staining cells. DAPI stains nuclei blue. Scale bars, 50 μm. Quantification of the number of p-VEGFR2^+^ cells per tissue area (N. p-VEGFR2^+^ cell per mm^2^; **e**). *n*=5. Data are represented as mean±s.e.m. *P<0.001 versus Sham. ^#^*P*<0.001 versus injury 4 W+Veh (vehicle) group as determined by ANOVA. (**f**) Schematic model indicating RhoA-MMP3-CTGF-VEGF axis in the fate decision of MSCs during arterial remodelling. Activation of RhoA stimulates CTGF expression and CTGF–VEGF complex formation in ECM, resulting in differentiation of MSCs into SMC/myofibroblasts (left panel); inactivation of RhoA promotes MMP3 production, which cleavages CTGF and releases VEGF from CTGF–VEGF complex, leading to endothelial differentiation of MSCs (right panel).

If MMP3 mediates the angiogenic effect elicited by TGFβ with simultaneous RhoA inactivation in MSCs, the expression level of MMP3 in MSCs after arterial injury should be negatively correlated to the activation of RhoA during arterial remodelling. Consistent with the *in vitro* results, increased CTGF expression and enhanced ECM formation, indicated by increased fibronection and collagen I expression, was observed at the neointimal tissue of the injured arteries in *Nestin-cre::ROSA26-EYFP* mice (Fig. 8a). MMP3 expression was detected in 70% of the arterial endothelial cells in sham mice but almost undetectable in the *Nestin-EYFP*^*+*^ cells on the endoluminal side at both 1 and 4 weeks after injury (Fig. 8b,c). Importantly, high levels of MMP3 were detected at the edge of the intima layer closest to the regenerated endothelial cells in fasudil-treated mice (Fig. 8b,c). Moreover, significantly increased p-VEGFR2 (Tyr1175), a direct downstream signalling activated after VEGF–VEGFR binding, was detected in EYFP^+^ cells at the endoluminal layer of the vessels from fasudil-treated mice (Fig. 8d,e; ANOVA), supporting specific-localization of VEGF release for reendothelization of the injured vessels.

## Discussion

Interactions between the cell and the ECM are critical in controlling the plasticity of stem cells. In the present study, we have identified RhoA/ROCK as a key determinant for the lineage fate of MSCs during their participation in the repair/remodelling of injured arteries. RhoA acts to favour VEGF sequestration versus activation/release in ECM. Specifically, after endothelial injury, TGFβ-stimulated RhoA activation promotes the secretion of CTGF and VEGF as well as CTGF–VEGF complex formation in ECM, leading to MSC myofibroblast differentiation (Fig. 8f, left panel). RhoA/ROCK inhibition produced greater amounts of MMP3, which were capable of degrading CTGF to release active VEGF from ECM, altering MSC differentiation towards endothelial lineage cells (Fig. 8f, right panel). Thus, the ‘on' and ‘off' switch of RhoA in MSCs directly modulated the ECM microenvironment, which in turn changed the lineage fate of MSCs after arterial injury.

The mouse model of endoluminal injury in arteries provides an ideal opportunity for the study of the involvement of MSCs in tissue repair/remodelling. Both our previous study^13^ and the present work reveal that the nestin^+^ cells can be recruited to the injured arteries, contributing to the formation of neointima. Although nestin^+^ cells are a mixed population, MSCs are the predominant component (80–90%). Parabiosis mouse model creates shared circulation system, in which approximately half of the partner-derived cells can be detected in the blood from the other partner due to blood chimerism^35^,^36^. The data from our parabiosis experiment revealed a similar percent of blood chimerism between the parabionts. After injury to the femoral artery in the WT mice of the heteroparabiotic pair, ∼22.5% of total neointimal cells engrafted into the neointima tissue were partner-derived EYFP^+^ cells. Considering the chimeric circulation with about half of nestin^+^ cells being labelled as EYFP^+^ in the Nes-R26-EYFP and WT heteroparabiotic pair, the parabiosis data suggest that up to 45% of the neointimal cells observed at the remodelling injured arterial sites are Nestin^+^ MSCs derived from distal sites via blood circulation. This estimated number is close to the result from the lineage mapping mouse model, in which we found 41% of the neointimal cells are EYFP^+^. In spite of the finding, we cannot exclude the possibility that some nestin^+^ cells in the neointima may also originate from local sites. We observed EYFP^+^ cells in the media layer of the injured arteries but not in the uninjured control arteries. It is possible that some smooth muscle cells in the vascular media may have dedifferentiated to nestin^+^ cells in response to the injury of the arteries. A previous lineage tracing study, using carotid artery ligation mouse model, reported that *Mhy11-* or *Acta*2-tagged medial vascular smooth muscle cells contribute to the majority of neointima formation, indicating that the cells derived from local but not distal sites play a primary role in arterial remodelling^37^. However, the contribution of bone marrow/circulating cells to vascular remodelling highly depends on the type of arterial injury^38^. It was demonstrated that after wire-mediated endovascular injury, a significant number of the neointimal cells were derived from bone marrow/blood circulation. In contrast, there were only a few bone marrow/circulating-derived cells in the neointima after ligation of the carotid artery^38^. Future study is needed to evaluate the distinct roles of local and circulating nestin^+^ cells in arterial remodelling using different animal models.

We previously showed that nestin^+^ MSCs were recruited from circulating blood to the injured sites to form neointima, mediated primarily by TGFβ-Smad-MCP1 signalling^13^. Here we found that TGFβ also activates RhoA signalling in the newly recruited nestin^+^ MSCs to drive their differentiation to neointimal SMCs/myofibroblasts. Thus, TGFβ, as an injury-activated messenger, regulates two different action modes of MSCs, that is, migration and differentiation via activating distinct signalling pathways, to participate in vascular remodelling. Our previous work revealed that the activation of TGFβ1 from latent form to free form was induced by the injury of vascular matrix using the same arterial injury mouse model^13^. Therefore, we consider injured artery wall as one of the major sources to release active TGFβ. Interestingly, SB505124, a selective inhibitor of TβRI serine/threonine kinase activity, inhibited both Smad2 phosphorylation and RhoA activation at a higher concentration, however, only inhibited Smad2 phosphorylation without affecting RhoA activation at a lower concentration. The mechanism underlying this phenomenon is unclear. Although most of the previous studies showed a TβRI/Smad-dependent mechanism for TGFβ-activated RhoA signalling^39^,^40^, a recent study reported a TβRII-Src signalling-mediated but TβRI/Smad-independent mechanism for TGFβ-stimulated early RhoA/RhoB activation^41^. The result from the current study indicates that RhoA activation in our model system may also involves TβRI/Smad-independent mechanisms. The finding that lower concentration of this inhibitor failed to inhibit MSC differentiation suggests that TGFβ stimulates MSC differentiation primarily through a RhoA-dependent pathway in our model system. Previous animal studies have documented other TGFβ downstream effectors, such as ERK1/2, JNK1/2 and p38α, involvement in neointima formation^42^. Our results from the injured aorta-CM-based migration and differentiation assays suggest that TGFβ-activated ERK1/2, JNK1/2 and p38α are less important for nestin^+^ MSC-involved vascular remodelling. As ∼40% of the neointimal cells originate from MSCs, presented here and found previously^43^, it is possible that the MAPK cascade induces the proliferation and transformation of the local medial SMCs into neointimal myofibroblasts, whereas RhoA signalling primarily mediates differentiation of recruited nestin^+^ MSCs. Thus, intimal hyperplasia is likely developed from the combined results of MAPK-stimulated proliferation of medial SMCs and RhoA-stimulated differentiation of recruited MSCs.

RhoA GTPase is a master regulator for the cytoskeleton tension and cell shape to modulate MSC lineage commitment and differentiation^44^,^45^,^46^. The direction of RhoA/ROCK-mediated MSC differentiation depends on specific stimuli or local cues. For example, RhoA/ROCK mediates cell shape change-induced MSC commitment towards osteogenic versus adipogenic fates^46^. Specifically, RhoA upregulation induced by the deficiency of a RhoA negative regulator p190RhoGAP leads to an adipogenic-myogenic switch of MSCs in response to IGF-1 exposure^47^. p190RhoGAP also regulates a switch between cardiomyogenic and endothelial lineage in response to mechanical signals^48^. Here, we have found that activation or inhibition of RhoA/ROCK switches the differentiation of MSCs between SMC/myofibroblast and endothelial cells, respectively, during arterial repair/remodelling and was mediated by specific MSC–ECM interactions. Thus, the different lineage directions of MSCs mediated by RhoA/ROCK likely depend on tissue-specific ECM composition. Following aorta injury, we found that TGFβ-mediated RhoA activation results in increased secretion of CTGF and VEGF, forming a complex deposited in the ECM. However, inhibition of RhoA led to higher MMP3 production, cleavage of CTGF, and VEGF release to trigger MSC differentiation to endothelial lineage. Further studies evaluating ECM-MSC contexts are needed to determine if RhoA-mediates similar osteogenic-adipogenic or adipogenic-myogenic switches. The function of RhoA/ROCK during angiogenesis and vascular remodelling remains debated as studies in cancer development have suggested that ROCK inhibition blocks angiogenesis^49^,^50^, whereas other reports, including ours, have shown RhoA/ROCK inhibition promotes angiogenesis and rescues endothelial cell dysfunction^51^,^52^,^53^,^54^. We speculate that the inconsistent results on the function of RhoA/ROCK in angiogenesis are mainly attributed to the relative levels and availability of distinct MMPs and different proteolytic targets of MMPs in the specific ECM microenvironments.

We found that MSCs differentiated into endothelial cells after the cells were incubated with TGFβ plus RhoA inhibitor or after the cells were transfected with active RhoA and treated with MMP3. In ROCK inhibitor-treated mice, the majority of the MSC-derived EYFP^+^ cells in the repaired endothelium after arterial injury were MMP3-positive but GTP-RhoA-negative, suggesting that MSCs are the major source of MMP3 secretion. MMP9, MMP14 or MMP2 has also been found to play a role in angiogenesis or vascular remodelling^55^,^56^. For example, MMP9 renders VEGF release and triggers the angiogenic switch during carcinogenesis of pancreatic islets in mice^57^. We did not detect any changes in other MMPs in DNA microarray of the active RhoA-overexpressed MSCs indicating a specific role of MMP3 in TGFβ/RhoA-regulated ECM remodelling. However, a more detailed investigation is needed to evaluate the possible involvement of other MMPs in this process. CTGF is also called CCN family member 2 (CCN2). In addition to CTGF/CCN2, we also observed changes of CCN5 and CCN3 level in the DNA microarray. CCN3 and 5 might also sequestrate VEGF in ECM in response to RhoA activation or be susceptible to MMP3 cleavage. We also notice that VEGF NAb could not completely block TGFβ plus ROCK inhibitor-stimulated tube formation of MSCs, indicating mechanisms other than MMP3-CTGF-VEGF axis may also be involved in the lineage switch of MSCs. It has been shown that four (Oct4, Klf4, Sox2 and c-myc)^58^ or two iPSC factors (Oct4 and Klf4)^59^ transdifferentiated fibroblasts to functional endothelial cells. It is possible that TGFβ plus RhoA inhibitor treatment first stimulates the expression of these factors to convert MSCs to endothelial progenitors, which is then targeted by VEGF.

Our study presents an interesting opportunity for the potential application of RhoA/ROCK inhibitors or transplantation approach using MMP3-overexpressed MSCs in patients with coronary atherosclerosis or post coronary angioplasty. It is known that early reendothelialization after the initial endothelium damage reduces stenosis in the arteries of these patients^15^,^16^,^17^. We found that a ROCK inhibitor fasudil almost completely restored the damaged endothelium layer and significantly reduced intimal hyperpolasia in the injured arteries of mice. Fasudil is a therapy for cerebral vasospasm^60^ and poses feasibility for future clinical trials. In addition, the repair/remodelling of other tissues and wound healing are often characterized by angiogenesis and the recruitment of fibroblasts. Fibroblasts synthesize ECM components and paracrine factors, including VEGF and CTGF. Our study identifies TGFβ-RhoA-MMP3-CTGF-VEGF axis as a molecular basis for reciprocal MSC–ECM interactions during arterial remodelling and provides a basis for expansion of studying further MSC-ECM pathways to identify targets in MSC-based cell therapy and tissue regeneration.

## Methods

### Animals and treatment

Sprague–Dawley rats and wild-type C57BL/6J mice were purchased from Charles River. *Nestin-GFP* mice were provided by Dr Grigori Enikolopov at Cold Spring Harbor Laboratory. *B6.Cg-Tg(Nes-cre)1Kln/J* mice (Stock No: 003771) and *B6.129X1-Gt(ROSA)26Sor*^*tm1(EYFP)Cos*^*/J* mice (Stock No: 006148) were purchased from Jackson Laboratory. Eight- to twelve-week-old male mice and 12-week-old male rats were used in this study. We treated the mice with Fasudil (LC Laboratories) at a dose of 30 mg kg^−1^ dissolved in water by an oral gavage twice daily for 4 weeks. Vehicle-treated mice received water. All animals were maintained in the animal facility of the Johns Hopkins University School of Medicine. The experimental protocol was reviewed and approved by the Institutional Animal Care and Use Committee of the Johns Hopkins University, Baltimore, MD, USA.

### Mouse model of wire-induced injury of femoral artery

Transluminal mechanical injury of the femoral artery was conducted as described previously^61^. Briefly, either the left or right femoral artery was subjected to blunt dissection. A straight spring wire (0.38 mm in diameter, No. C-SF-15-15, COOK) was inserted towards the iliac artery to reach 5-mm distance from the incision. The wire was left in place for 1 min to denude endothelium and dilate the artery. Blood flow in the femoral artery was restored by releasing the sutures placed in the proximal and distal portions. At 1 or 4 weeks after surgery, mice were killed by i.p. administration of ketamine/xylazine. The femoral artery was excised, fixed with 4% paraformaldehyde and embedded in paraffin. Thin sections (5 μm) were used for histological and immunohistochemical analyses.

### Parabiosis

Parabiotic surgery was performed as described previously^62^. A male *Nestin-Cre; C57BL/6J-Gtrosa26 tm1EYFP* mice was surgically joined to a male *C57BL/6J* mice (Heterochronic parabiotic pairs). *Nestin-Cre; C57BL/6J-Gtrosa26 tm1EYFP* mice conjoined to *Nestin-Cre; C57BL/6J-Gtrosa26 tm1EYFP* mice and *C57BL/6J* mice conjoined to *C57BL/6J* mice served as control (Isochronic parabiotic pairs). Briefly, 2-month-old mice were anaesthetized. The hair of the dorsal and lateral aspects was removed and matching skin from the shoulder to the knee joint of each mouse was incised. Approximate 1 cm incisions in the peritoneum were made in each mouse, and the mice were attached using 3-0-coated Vicryl (Ethicon). The dorsal and ventral skin were stitched through continuous suture. Individual parabiotic mouse pairs were placed in clean cages, and food pellets were provided on the floor to minimize the strain of reaching for food while adjusting to parabiotic existence. Established shared blood circulation was confirmed by flow cytometry analysis of EYFP^+^ cells in the blood from parabiotic mouse at 2 weeks post surgery^63^. After the cross-circulation of the two mice was confirmed, wire-induced injury of femoral artery was performed to the *C57BL/6J* mice (as described above). Arterial tissue from the *C57BL/6J* mice of parabiotic pair were collected 4 weeks after injury.

### Histological analysis of neointima

To study the morphology of the arteries, vessels were perfused with PBS followed by 4% paraformaldehyde by cannulating the left ventricle. Five-micrometre sections were stained with haematoxylin and eosin. Morphometric analysis of neointima formation consisted of the measurement of intimal area (*I*), medial area (*M*) and *I*/*M* ratios with a computerized morphometric analysis system (Image Pro Plus 6.0, Olympus) by an investigator blinded to the treatment. For each artery section, five random, noncontiguous microscopic fields were examined. All measurements performed on the four sections of the artery were averaged.

### Double-immunofluorescence analysis of the vessel sections

Double immunofluorescence staining was performed as described previously^64^,^65^. After blocking in 0.5% horse serum, sections were incubated with first antibodies anti-GFP (Rockland, 600-101-215,1:500, or Abcam, ab290,1:200), anti-Nestin (Aves Labs, NES, 1:100), anti-active RhoA-GTP (NewEast Bioscience, 26904, 1:100), anti-MMP3 (Abcam, ab52915, 1:100), anti-CD31 (Abcam, ab28364, 1:50), anti-αSMA (Abcam, ab5694, 1:200), anti-CD11b (Abcam, ab8878, 1:200), anti-VE-cadherin (Abcam, ab33168, 1:100), anti-Leptin Receptor (R&D, BAF497, 1:200), anti-fibronectin (Abcam, ab2413, 1:200), anti-collagen I (Abcam, ab21286, 1:200), anti-phospho-VEGFR2 (Tyr1175) (Abcam, ab38464, 1:100), followed by incubation with FITC or Cy3-conjugated secondary antibodies (Jackson ImmunoResearch). Nuclei were counterstained with 4,6-diamidino-2-phenylindole (Sigma). The sections were mounted with the ProLong Antifade Kit (Molecular Probes) and observed under a confocal microscope (FLUOVIEW FV300, Olympus).

### Cell sorting and flow cytometry analysis

Blood samples were collected from mice by cardiac puncture. After the process of RBC lysis with commercial ACK lysis buffer (Quality Biological, Inc), cells were washed with 0.1% BSA in PBS. Cells were then sorted according to side scatter and GFP expression after negative selection of leukocyte common antigen CD45. Fluorescence-activated cell sorting (FACS)-sorting was performed using a five-laser BD FACS and FACSDiva. Flow cytometric analyses were carried out using a FACSCalibur flow cytometer and CellQuest software (Becton Dickinson). After RBC lysis of the blood samples, the cells were counted. The primary antibodies used were FITC-conjugated anti-GFP, PE-conjugated anti-Sca-1, APC-conjugated anti-CD31/PECAM-1, PerCP-conjugated anti-CD45 and PerCP-conjugated anti-CD105. These antibodies are all commercially available from Bio-Legend. Antibody against Leptin Receptor was purchased from R&D, followed by incubation with Cy3-conjugated secondary antibody.

### Characterization of nestin^+^ cells

Blood samples were harvested from *Nestin-GFP* mice at 1 week after arterial injury. GFP^+^LepR^+^ cells and GFP^+^LepR^−^ were sorted by FACS. The sorted cells were enriched, cultured and further confirmed by flow cytometry. For osteogenic differentiation, cells were seeded at a density of 5 × 10^3^ per cm^2^ with αMEM supplemented with 10% fetal bovine serum, 0.1 μM dexamethasone, 10 mM β-glycerol phosphate and 50 μM ascorbate-2-phosphate. After 3 weeks of differentiation, the mineralization capacity of the cells was evaluated by Alizarin Red staining. For adipogenic differentiation, cells were seeded at a density of 1 × 10^4^ per cm^2^ with α-MEM supplemented with 10% fetal bovine serum, 1 μM dexamethasone, 0.5 μM 3-isobutyl-1-methylxanthine and 10 ng ml^−1^ of insulin for 2 weeks. Lipid accumulation was identified by oil red O staining. For chondrogenic differentiation, cells (1 × 10^6^) were seeded in polypropylene tubes with high-glucose D-MEM supplemented with 0.1 μM dexamethasone, 1% insulin-transferrin-sodium selenite mix, 50 μM ascorbate-2-phosphate,1 mM sodium pyruvate, 50 μg ml^−1^ of proline and 20 ng ml^−1^ of TGF-β3. After 3 weeks in culture, the pellets were fixed in 10% buffered formalin for 2 days and embedded in paraffin. Then 4-μm-thick sections were processed for toluidine blue staining. The reagents are all commercially available from Sigma-Aldrich. For smooth muscle cell and endothelial cell differentiation, cells were seeded at a density of 1 × 10^4^ per cm^2^ and cultured with smooth muscle cell differentiation medium (ThermoFisher Scientific, S-008-5) and endothelium differentiation medium (Celprogen, M66110-37DS) for 7 days, respectively, followed by immunofluorescence staining with antibodies against α-SMA and CD31.

### Cells and gene transfection

Mouse bone marrow MSCs were purchased from Texas A&M Health Science Center College of Medicine Institute for Regenerative Medicine. All MSCs were cultured in αMEM supplied with 10% FCS. The transfection of DNA plasmids were performed with Lipofectamine LTX with Plus Reagent (Life Technologies). The transfection of siRNA was performed with Lipofectamine RNAiMAX Reagent (Life Technologies).

### Aorta-CM-based assays

To prepare vascular-CM, male Sprague–Dawley rats (250–300 g) were perfused with 200 ml of 0.9% saline and their ascending thoracic aortae were isolated from the peri-adventitial tissue under a dissection microscope. The isolated aortae were cut into three pieces with equal length and were flushed with sterilized phosphate-buffered saline (PBS). Endothelium injury was achieved by rubbing the luminal surface three times with a cotton-tipped applicator as described previously^66^. Each segment of injured aorta and the uninjured control were cultured in 24-well tissue culture plates with 600 μl serum-free DMEM at 37 °C. Two days later, the CM were collected and stored at −80 °C. In some experiments, neutralizing antibodies against TGFβ1 or various compounds were individually added to the CM. Myofibroblasts/SMCs differentiation of MSCs were performed by incubating mouse bone marrow MSCs with αMEM, Uninjured aorta-CM or injured aorta CM prepared as described above for 7 days. Protein extracts were isolated and subjected to western blot analysis using specific antibody against myofibroblast/SMC marker αSMA. In a separate set of experiments, MSCs were cultured in a coverslip chamber in αMEM, uninjured aorta-CM or injured aorta-CM prepared as described above for 7 days. Cells were fixed with 4% paraformaldehyde and permeabilized by Triton X-100. After blocking in 0.5% horse serum, sections were incubated with the primary antibody followed by incubation with Cy3-conjugated secondary antibodies (Jackson ImmunoResearch). Nuclei were counterstained with 4,6-diamidino-2-phenylindole. The sections were mounted with the ProLong Antifade Kit (Molecular Probes) and observed under a fluorescence microscope (FLUOVIEW FV300, Olympus). In addition, RNAs were isolated from the cells, and the mRNA levels of CTGF and Col1A were measured by quantitative PCR.

### RNA and microarray processing

Myc-L63RhoA or EV was transfected into mouse bone marrow MSCs. Three days later, the cells were harvested and total RNA was isolated using RNeasy Mini kit (Qiagen). RNA samples were assessed for quality and integrity using Synergy HT (Biotek). Microarray expression profiles were generated using the Illumina MouseRef-8 v2.0 Expression BeadChip (Illumina). Biotin-labelled cRNA was synthesized by the total prep RNA amplification kit from Ambion. cRNA was quantified and normalized to 75 ng μl^−1^, and then 1 μg was hybridized to Beadchips. The hybridized chip was scanned using the Illumina iScan system and background corrected signal intensities were extracted using the GenomeStudio software (Illumina). The lumi R package was used to transform the data using a Variance Stabilizing Transformation and normalized using quantile normalization^67^. Differential expression analysis was performed using the R/Mannova package. *P*-values were calculated by performing 1,000 permutations, then corrected for multiple comparisons by false-discovery rate transformation, using a 20% false-discovery rate cutoff^68^.

### Transcriptional validation

Primers (Supplementary Table 1) were designed for candidate genes using primer bank database and Primer3 (v.0.4.0) software. RNA was reverse transcribed to cDNA using SuperScript III First Strand Synthesis System for RT–PCR (Invitrogen) according to the manufacturer's protocols. qRT–PCR was performed using SYBR Green PCR Master Mix and 7900 HT Fast Real-Time PCR System (Applied Biosystems). Each reaction was run in triplicate and consisted of 100 ng of cDNA, 1–50 μM optimized primers and 1 × Fast Power SYBR Green Mix. Amplification efficiency was determined using serially diluted endometrial epithelial cDNA (200-0.2 ng) using the slope of best fit curve for cycle threshold and concentration. The amplification conditions were 95 °C for 10 min, 95 °C for 15 s and 60 °C for 1 min. No-template and no-RT controls were included for each assay to ensure quality and cDNA specificity of the primers. PCR products were verified by agarose gel electrophoresis. Target gene expression was normalized to GAPDH mRNA and relative gene expression assessed using the 2^−ΔΔCT^ method^69^.

### ECM protein extraction

After different treatment, cells were washed with cold PBS and cross-linked with 5 mM BS3 (bis(sulfosuccinimidyl)suberate) for 30 min at room temperature. Cross-link was stopped by addition of 50 mM (final concentration) Tris buffer (pH 7.5) for 15 min at room temperature. We lysed and discarded cells by three successive treatments with ice-cold hypotonic 1:5 diluted PBS containing 0.5% (w/v) of DOC (Sodium Deoxycholate). ECM proteins were scrapped from the dish with lysis buffer: 1% Triton X-100, 10% glycerol, 5% SDS, 100 mM dithiothreitol and 1% protease inhibitor cocktail set^70^. Samples were heated 10 min at 90 °C. Protein amounts were estimated using the Protein Assay (Bio-Rad).

### Tube formation assay

50 μl per well Matrigel (BD Biosciences) was plated in 96-well culture plates and incubated at 37 °C to polymerize for 1 h. Then 2 × 10^4^ per well MSCs were seeded on polymerized Matrigel in plates. The cells were cultured with different overlay media collected from MSCs with various treatments. After 4 h of incubation at 37 °C, tube formation was visualized under microscopy and cumulative tube lengths were measured.

### Immunoprecipitation and western blot analysis

Immunoprecipitation were performed as described previously^64^,^65^. Briefly, different overlay media collected from MSC cultures of different treatments were concentrated and immunoprecipiated by incubation with the VEGF antibody (Abcam, ab69479, 1:50), followed by adsorption to protein G Sepharose. Immunoprecipitates, overlay media protein and ECM protein were separated by SDS–PAGE, blotted onto a polyvinylidene difluoride (Bio-Rad Laboratories) membrane. The membrane was incubated with antibodies against: CTGF (Abcam, ab6992, 1:5,000), VEGF (Abcam, ab69479, 1:1,000), MMP3 (Abcam, ab52915, 1:1,000) and visualized by enhanced chemiluminescence (ECL Kit; Amersham Biosciences). The uncropped scans of blots are shown in Supplementary Figs 9 and 10.

### Statistics

Data presented as mean±s.e.m. The unpaired, two-tailed Student's *t*-tests was used for comparisons between two groups. For multiple comparisons, one-way ANOVA with Bonferroni *post hoc* test was applied. All data demonstrated a normal distribution and similar variation between groups. Statistical analysis was done using SAS software. *P* values<0.05 were deemed significant.

## Additional information

**Accession codes**: Microarray data have been deposited in the GEO database under accession code: GSE73557.

**How to cite this article:** Li, C. *et al.* RhoA determines lineage fate of mesenchymal stem cells by modulating CTGF–VEGF complex in extracellular matrix. *Nat. Commun.* 7:11455 doi: 10.1038/ncomms11455 (2016).

## Supplementary Material

Supplementary Information Supplementary Figures 1-10 and Supplementary Table 1
