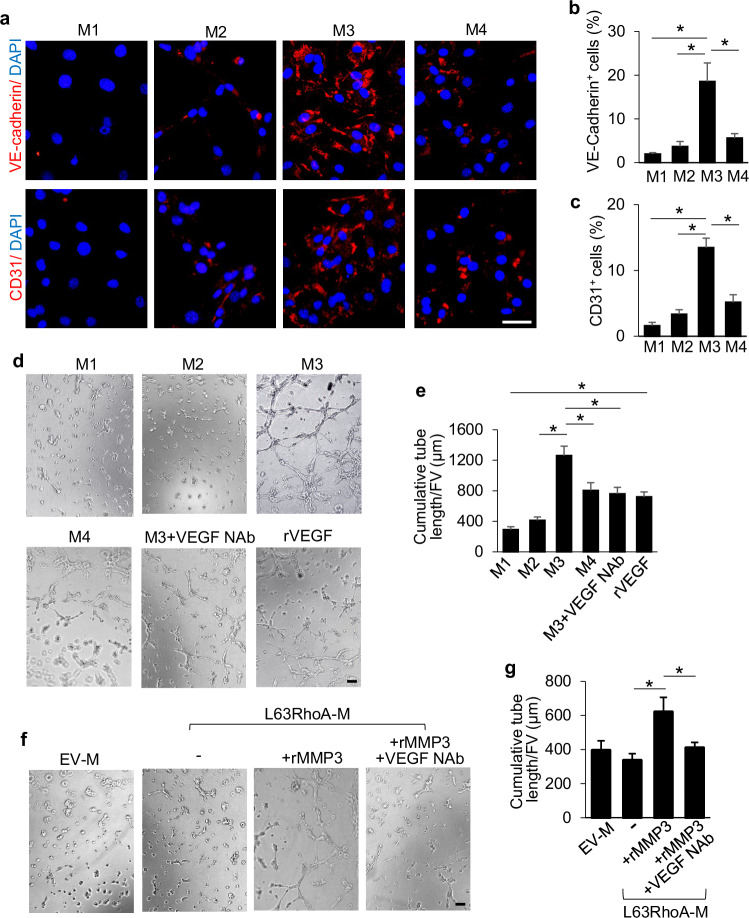# Author Correction: RhoA determines lineage fate of mesenchymal stem cells by modulating CTGF–VEGF complex in extracellular matrix

**DOI:** 10.1038/s41467-025-61274-3

**Published:** 2025-07-02

**Authors:** Changjun Li, Gehua Zhen, Yu Chai, Liang Xie, Janet L. Crane, Emily Farber, Charles R. Farber, Xianghang Luo, Peisong Gao, Xu Cao, Mei Wan

**Affiliations:** 1 Department of Orthopaedic Surgery, Johns Hopkins University School of Medicine, Baltimore, Maryland 21205 USA; 2 Department of Public Health Sciences, Center for Public Health Genomics, University of Virginia, Charlottesville, Virginia 22908 USA; 3 Department of Endocrinology, Institute of Endocrinology and Metabolism, Second Xiangya Hospital of Central South University, Changsha, 410011 China; 4 Department of Medicine, Johns Hopkins Asthma and Allergy Center, Johns Hopkins University School of Medicine, Baltimore, Maryland 21224 USA

Correction to: *Nature Communications* 10.1038/ncomms11455, published online 29 April 2016

In the version of the article initially published, the “M2” image in Fig. 7d was incorrect due to an error in figure preparation. The correct image has been located, and the amended figure can be seen as Fig. 1, below. This notice serves to correct the error.

Fig. 1 Corrected Fig. 7