# Supplementary material for: RhoA determines lineage fate of mesenchymal stem cells by modulating CTGF–VEGF complex in extracellular matrix
Source: Nat Commun. 2016 Apr 29;7:11455. doi: 10.1038/ncomms11455 (PMC4855537; doi:10.1038/ncomms11455)
Supplement: Supplementary Information — Supplementary Figures 1-10 and Supplementary Table 1 [file ncomms11455-s1.pdf]

Supplementary Fig. 1

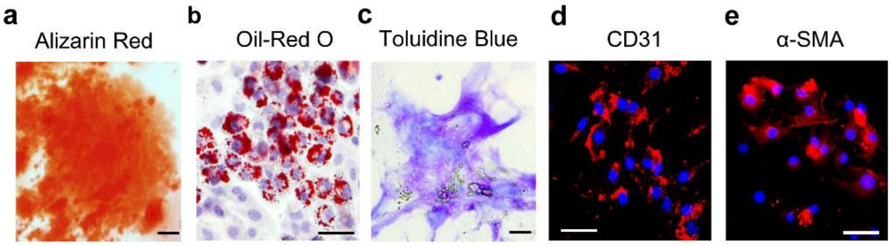

Supplementary Figure 1. Nestin-GFP$^+$ cells have multiple-differentiation capacity. Nestin-GFP mice were subjected to wire insertion-induced injury in femoral arteries. Blood samples were collected 1 week later, and the CD45$^-$GFP$^+$LepR$^+$ cells were subjected to FACS sorting. The sorted cells were then cultured in single colony, and individual colonies were selected and expanded by passaging. Each clonal strain derived from a single CFU-F underwent osteogenic (**a**, Alizarin red stain), adipogenic (**b**, Oil-red O stain), chondrogenic (**c**, Toluidine blue stain), angiogenic (**d**, CD31 immunostaining) and myofibroblastic (**e**, α-SMA immunostaining) induction by incubation with various specific differentiation medium. Scale bars: 50μm.

Supplementary Fig. 2

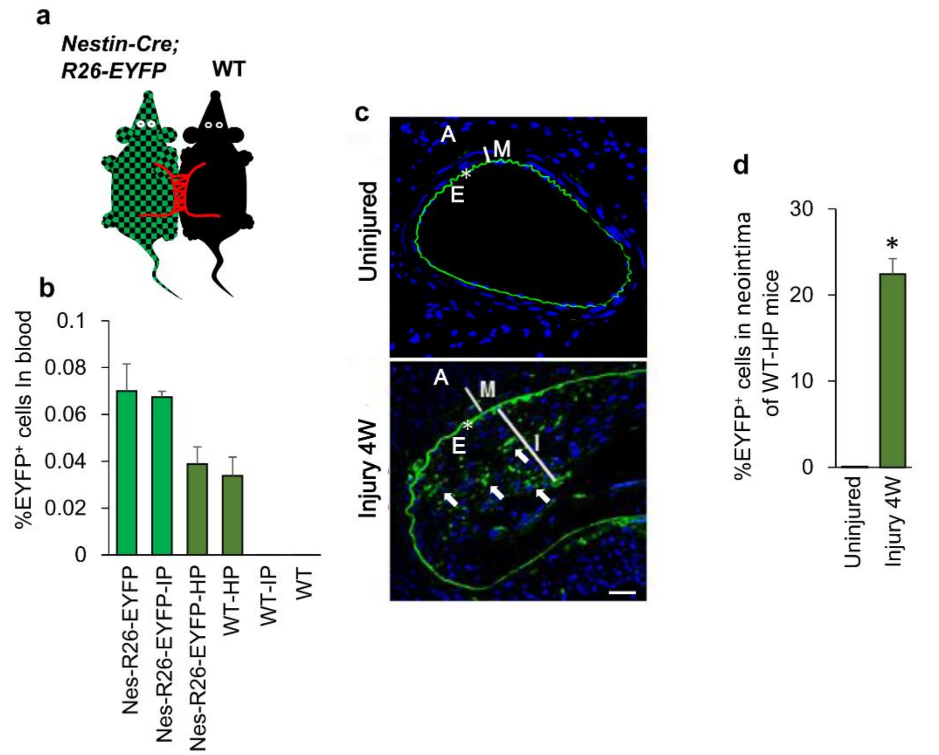

Supplementary Figure 2. Circulating nestin⁺ cells contributed to neointima formation after arterial injury.

(**a**) Schematic model of a heteroparabiotic parabiosis mouse pair (HP). Injury was induced in the left femoral artery of WT mouse (WT-HP) that were surgically jointed to *Nestin-Cre; R26-EYFP* mouse (Nes-R26-EYFP-HP).

(**b**) Flow cytometry analysis of EYFP⁺ cells in circulating blood in mouse at the indicated groups. Nes-R26-EYFP-IP: Isochronic parabiotic pairs of *Nestin-Cre; R26-EYFP* mouse; WT-IP: Isochronic parabiotic pairs of WT mouse. n=5, Data are represented as mean ± s.e.m.

(**c**)The right (uninjured) and left (injured) arteries of WT-HP mouse were harvested at 4 weeks after the injury. EYFP⁺ cells in arteries were detected by immunofluorescence staining. I, intima layer; M, media smooth muscle layer; A, adventitia layer; E*, elastic fiber. White arrows represent positive cells. Scale bars=100 μm.

(**d**) Percentage of EYFP⁺ cells in neointima tissue in injured and uninjured arteries of WT-HP mouse. n=4, Data are represented as mean ± s.e.m. *p<0.001 as determined by Student's *t*-tests.

Supplementary Fig. 3

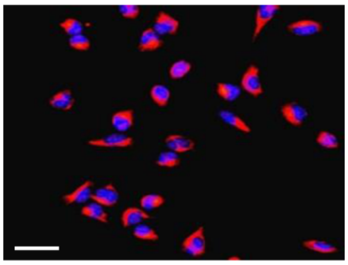

Supplementary Figure 3. Immunofluorescence staining of mouse marrow mesenchymal stem cells (MSCs) with antibody against Nestin (red). DAPI stains nuclei blue. Scale bars: 50μm.

Supplementary Fig. 4

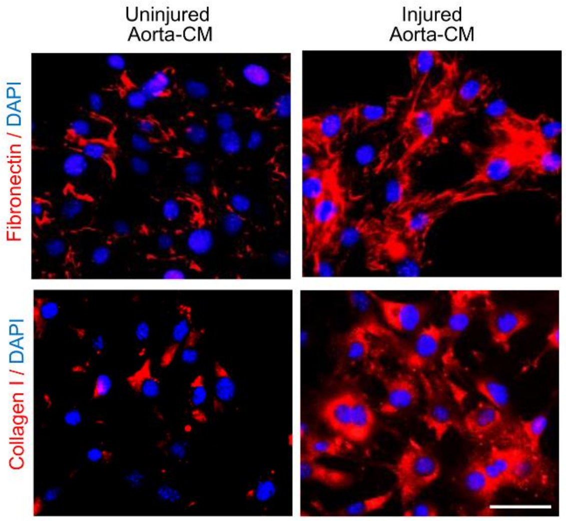

Supplementary Figure 4. Injured Aorta-CM promoted ECM formation in MSC culture.
Mouse MSCs were plated on pre-coated coverslips with Polylysine and incubated with Uninjured Aorta-CM or Injured Aorta-CM in 6-well plate for 7 days. Cells were fixed and not permeabilized by Trixon X-100. Immunofluorescence staining was performed using individual antibodies against fibronectin or collagen I (shown in red). DAPI stains nuclear blue. Scale bars: 50μm.

Supplementary Fig. 5

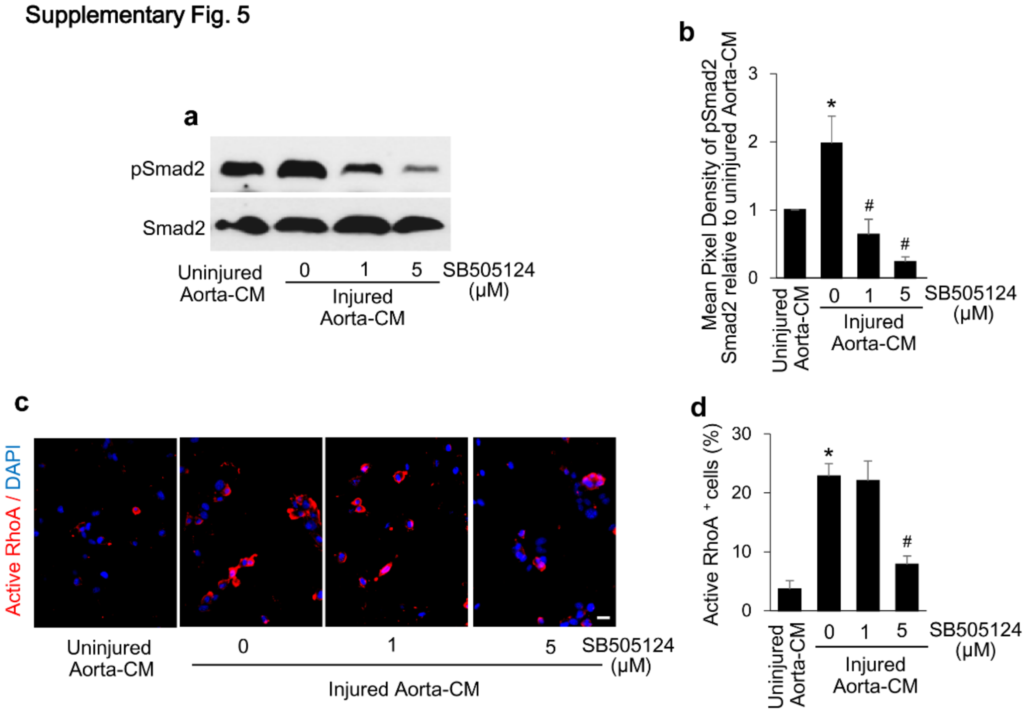

Supplementary Figure 5. High concentration of TβRI inhibiitor SB505124 inhibited both Smad and RhoA signalings, whereas low concentration of the inhibitor only inhibited Smad signaling.
(**a** and **b**) Western blot analysis of the effect of SB505124 on Injured Aorta-CM induced Smad2 phosphorylation. MSCs were incubated with Uninjured Aorta-CM or Injured Aorta-CM with addition of increasing doses of SB505124 for 2 hours. Western blot analysis of the cell lysates was performed using antibody against phophorylated Smad2 (pSmad2) and total Smad2 (**a**). Densitometric quantification of the density of each pSmad2 band and normalized by the corresponding total Smad2 band density (**b**). n=3 independent experiments; (**c** and **d**) Immunofluorescence analysis of the cells using antibody specifically against active RhoA-GTP. Representative images are shown (**c**). Percentages of total cells expressing active RhoA-GTP per 20X magnification field were calculated (**d**). n=4, Data are represented as mean ± s.e.m. *$p < 0.001$ vs. Uninjured aorta-CM, #$p < 0.01$ vs. Injured aorta-CM + Vehicle as determined by ANOVA. Scale bars: 50μm.

Supplementary Fig. 6

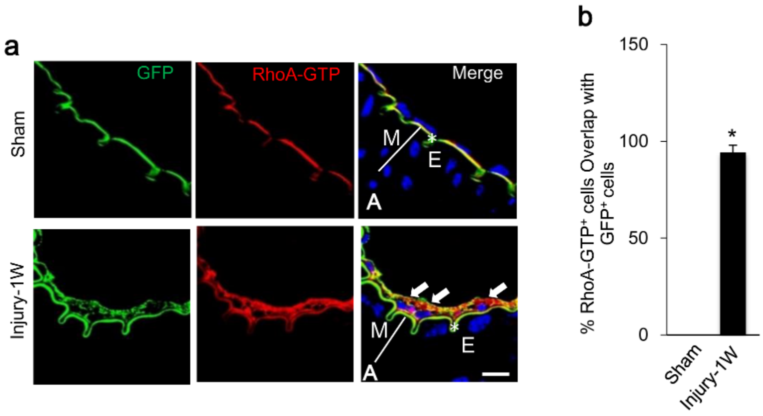

Supplementary Figure 6. The majority of nestin-GFP⁺ cells at the neointimal tissue expressed active RhoA-GTP.

Nestin-GFP mice were subjected to either sham surgery or wire insertion-induced injury in femoral arteries. One weeks after procedure, femoral artery samples were harvested. Double-immunofluorescence analysis of the artery sections was performed using antibodies against GFP and active RhoA-GTP. (**a**) Representative images were shown. GFP shows in green, and active RhoA-GTP shows in red. DAPI stains nuclei blue. White arrows represent double positive staining cells. I, intima layer; M, media smooth muscle layer; A, adventitia layer; E, elastic fiber. Scale bars: 50μm. (**b**) Quantification of the percentage of GFP^+ cells co-expressing active RhoA-GTP. n = 5. Data are represented as mean ± s.e.m. *p<0.001 vs. Sham as determined by Student's *t*-tests.

Supplementary Fig. 7

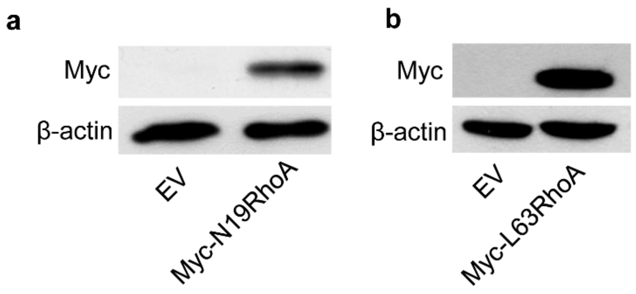

Supplementary Figure 7. Western blot analysis of MSCs transfected with N19RhoA or L63RhoA.

Mouse bone marrow MSCs were transfected with empty vector (EV), myc-tagged N19RhoA (Myc-N19RhoA) (**a**), or myc-tagged L63RhoA (Myc-L63RhoA) (**b**). Western blotting was performed to detect the protein expression using antibody against Myc. β-actin expression serves as a control.

Supplementary Fig. 8

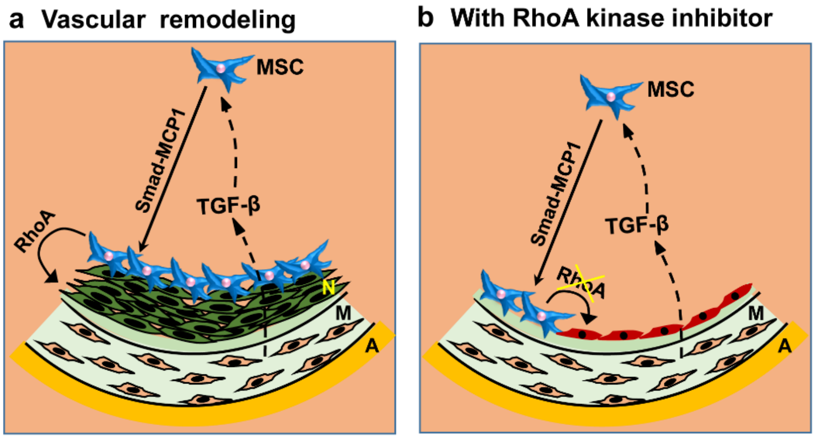

Supplementary Figure 8. Hypothetical model indicating the role of TGF-β and RhoA in recruitment and differentiation of MSCs after arterial injury.

(**a**) Active TGF-β, released from the injured arteries, stimulates the migration of MSCs to the injury site via Smad-MCP1 signaling. TGF-β also activates RhoA signaling to induce the differentiation of MSCs to SMCs/myofibroblasts, resulting in neointima formation.

(**b**) Inhibition of RhoA diverts the commitment/differentiation of MSCs away from SMCs/myofibroblasts toward endothelial cells, leading to endothelium repair.

N, neointima; M, media; A, adventitia.

Supplementary Fig 9. Uncropped scans of blots

Fig. 6e

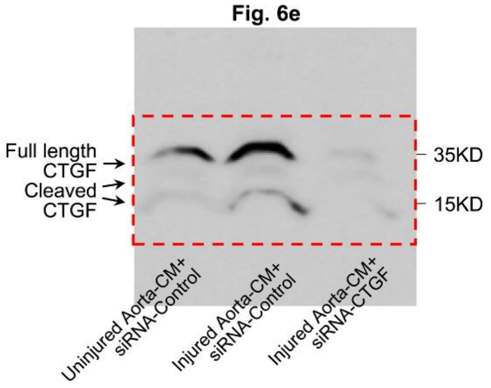

Fig. 6e

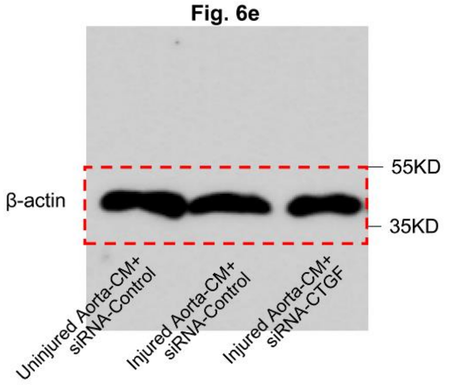

Fig. 6g

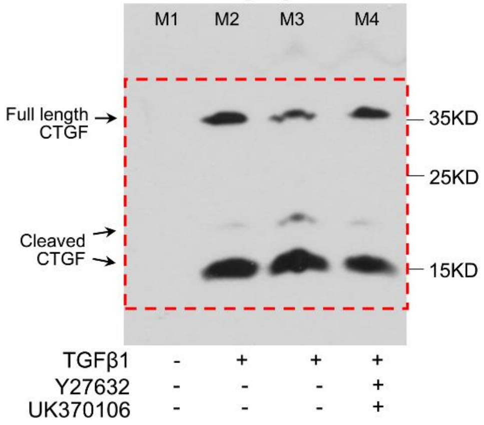

Fig. 6h

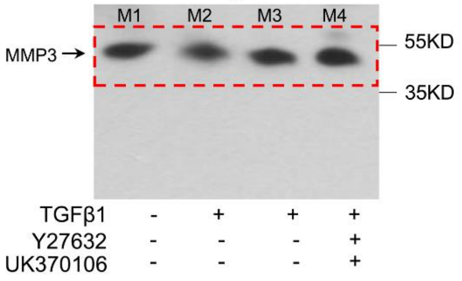

Fig. 6i

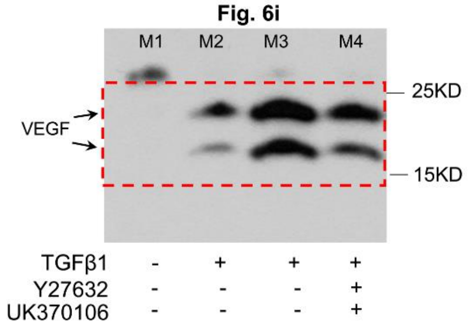

Fig. 6j

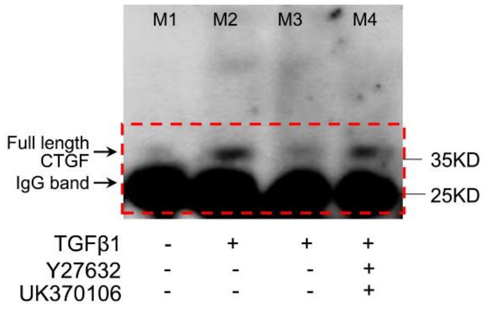

Supplementary Fig 10. Uncropped scans of blots

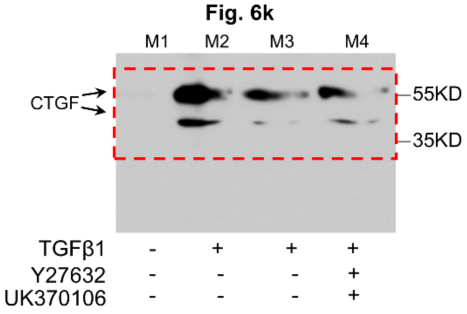

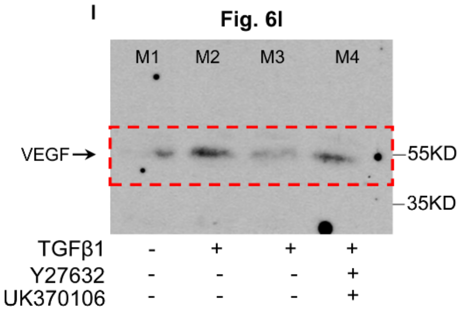

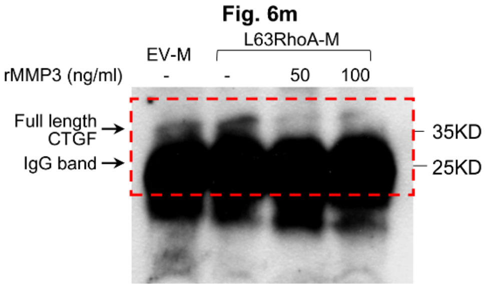

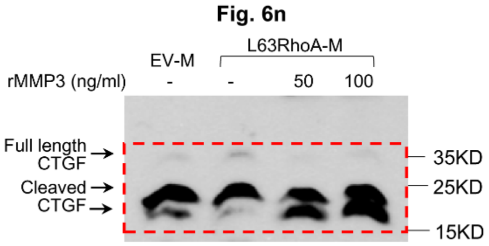

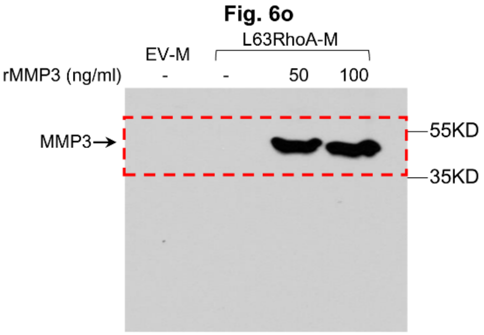

Supplementary Table 1. Primers used for quantitative real-time PCR

Primers	Forward	Reverse
CTGF	GGGCCTCTTCTGCGATTTC	ATCCAGGCAAGTGCATTGGTA
Col1A	GCTCCTCTTAGGGGCCACT	CCACGTCTCACCATTGGGG
Acta2	GTCCCAGACATCAGGGAGTAA	TCGGATACTTCAGCGTCAGGA
Mgp	TCCCGAAAGATTGGGCAAAAA	AAATGCACGGGCTAGGGTG
Wisp2	CGCTGTGATGACGGTGGTTT	CCTGGCACCTGTATTCTCCTG
Gsta1	AAGCCCGTGCTTCACTACTTC	GGGCACTTGGTCAAACATCAAA
Gsta4	TGATTGCCGTGGCTCCATTTA	CAACGAGAAAAGCCTCTCCGT
MMP3	ACATGGAGACTTTGTCCCTTTTG	TTGGCTGAGTGGTAGAGTCCC
TIMP3	CTTCTGCAACTCCGACATCGT	GGGGCATCTTACTGAAGCCTC
